# ricu: R’s interface to intensive care data

**DOI:** 10.1093/gigascience/giad041

**Published:** 2023-06-15

**Authors:** Nicolas Bennett, Drago Plečko, Ida-Fong Ukor, Nicolai Meinshausen, Peter Bühlmann

**Affiliations:** Seminar for Statistics, ETH Zürich, 8092 Zürich (Rämistrasse 101), Switzerland; Seminar for Statistics, ETH Zürich, 8092 Zürich (Rämistrasse 101), Switzerland; Department of Anaesthesiology and Perioperative Medicine, Monash Health, Clayton VIC 3168, Australia; Seminar for Statistics, ETH Zürich, 8092 Zürich (Rämistrasse 101), Switzerland; Seminar for Statistics, ETH Zürich, 8092 Zürich (Rämistrasse 101), Switzerland

**Keywords:** intensive care medicine, electronic health records, computational methods

## Abstract

**Objective:**

To develop a unified framework for analyzing data from 5 large publicly available intensive care unit (ICU) datasets.

**Findings:**

Using 3 American (Medical Information Mart for Intensive Care III, Medical Information Mart for Intensive Care IV, electronic ICU) and 2 European (Amsterdam University Medical Center Database, High Time Resolution ICU Dataset) databases, we constructed a mapping for each database to a set of clinically relevant concepts, which are grounded in the Observational Medical Outcomes Partnership Vocabulary wherever possible. Furthermore, we performed synchronization in the units of measurement and data type representation. On top of this, we built functionality, which allows the user to download, set up, and load data from all of the 5 databases, through a unified Application Programming Interface. The resulting ricu R-package represents the computational infrastructure for handling publicly available ICU datasets, and its latest release allows the user to load 119 existing clinical concepts from the 5 data sources.

**Conclusion:**

The ricu R-package (available on GitHub and CRAN) is the first tool that enables users to analyze publicly available ICU datasets simultaneously (datasets are available upon request from respective owners). Such an interface saves researchers time when analyzing ICU data and helps reproducibility. We hope that ricu can become a community-wide effort, so that data harmonization is not repeated by each research group separately. One current limitation is that concepts were added on a case-to-case basis, and therefore the resulting dictionary of concepts is not comprehensive. Further work is needed to make the dictionary comprehensive.

**Key points**

- The ricu R-package is the first tool that enables users to analyze 5 large intensive care unit datasets through a unified interface.
- ricu currently supports 119 clinical concepts across Medical Information Mart for Intensive Care IIII, Medical Information Mart for Intensive Care IV, electronic intensive care unit, High Time Resolution ICU Dataset, and Amsterdam University Medical Center Database datasets.
- ricu allows for easy addition of user-specified concepts and datasets, integrating them with the existing infrastructure.

## Introduction

Collection of electronic health records has seen a significant rise in recent years [1], opening up opportunities and providing the grounds for a large body of data-driven research oriented toward helping clinicians in decision-making and therefore improving patient care and health outcomes [2]. While growing amounts of collected patient data might not be easily utilized by intensivists for decision-making [3], this poses an opportunity for the application of machine learning (ML) methods.

One example of a problem that has received much attention from the ML community is early prediction of sepsis in the intensive care unit (ICU) [4–7]. Interestingly, there is evidence that a large proportion of the publications on this topic are based on the same dataset [8], the Medical Information Mart for Intensive Care III (MIMIC-III) [9], showing a systematic lack of external validation. This issue has recently again been highlighted by a study demonstrating poor performance in external validation of a widely adopted sepsis prediction model [10].

Contributing to this problem might well be the lack of computational infrastructure for handling multiple datasets. The MIMIC-III dataset consists of 26 different tables containing about 20 GB of data. While much work and care have gone into data preprocessing in order to provide a self-contained ready-to-use data resource with MIMIC-III, seemingly simple tasks such as computing a Sepsis-3 label [11] remain nontrivial efforts. (There is considerable heterogeneity in the number of patients satisfying the Sepsis-3 criterion [11] among studies investigating MIMIC-III. Reported Sepsis-3 prevalence ranges from 11.3% [4], over 23.9% [5] and 25.4% [12], and up to 49.1% [13]. While some of this variation may be explained by differing patient inclusion criteria, differences in label implementation must also contribute significantly.) This is only exacerbated when aiming to cointegrate multiple different datasets of this form, spanning hospitals and even countries, in order to capture effects of differing practice and demographics.

Given the somewhat specific focus of ICU data, it may come as a surprise as to how heterogeneous the currently available datasets are. In MIMIC-III and High Time Resolution ICU Dataset (HiRID), for example, time stamps are reported as absolute times (albeit randomly shifted due to data privacy concerns), whereas electronic ICU (eICU) and Amsterdam University Medical Center Database (AmsterdamUMCdb) use relative times (with origins being admission times). Another example involves different types of patient identifiers and their use among datasets. Common to all is the notion of an ICU admission identifier (ID), but apart from that, the amount of available information varies: while ICU (and hospital) readmissions for a given patient can be identified in some, this is not possible in other datasets. Furthermore, use of identifier systems might not be consistent over tables. In MIMIC-III, for example, some tables refer to ICU stay IDs while others use hospital stay IDs, which slightly complicates data retrieval for a fixed ID system. Additionally, table layouts vary (*long* versus *wide* data arrangement) and data organization in general is far from consistent over datasets.

In light of the above-described background, the aim of ricu is to provide computational infrastructure allowing users to investigate complex research questions in the context of critical care medicine as easily as possible, by introducing a unified interface to a heterogeneous set of data sources. The package enables users to write dataset-agnostic code that can simplify implementation and shorten the time necessary for prototyping code-querying different datasets. In its current form, the package handles 5 large-scale, publicly available intensive care databases out of the box: MIMIC-III [9] from the Beth Israel Deaconess Medical Center (BIDMC) in Boston, Massachusetts; the eICU Collaborative Research Database [14], containing data collected from 208 hospitals across the United States; HiRID [15] from the Department of Intensive Care Medicine of the Bern University Hospital, Switzerland; AmsterdamUMCdb [16] from the Amsterdam University Medical Center; and MIMIC-IV [17], again using data from BIDMC. Furthermore, ricu was designed with extensibility in mind, such that adding new public and/or private user-provided datasets is possible. Being implemented in R, a programming language popular among statisticians and data analysts, it is our hope to contribute to accessible and reproducible research by using a familiar environment and requiring only few system dependencies, thereby considerably simplifying setup.

To our knowledge, infrastructure that provides a common interface to multiple ICU datasets is a novel contribution. While there have been efforts [18, 19] attempting to abstract away some specifics of a dataset, these have so far exclusively focused on MIMIC-III, the most popular of public ICU datasets, and have not been designed with dataset interoperability in mind. It is also worth mentioning software packages ROMOP [20] and PatientExploreR [21] that offer some useful infrastructure for data compatible with the Observational Medical Outcomes Partnership (OMOP) common data model, but the heterogeneity of the datasets supported by ricu limits the ability to organize them according to such a data model.

## Findings

The ricu package can be accessed from either GitHub or CRAN, and the lastest development version can be installed by running:

devtools::install_github("eth-mds/ricu")

Alternatively, the package can be installed from CRAN by using the install.packages() command. Using ricu, the user can download and set up the 5 large ICU datasets from North America and Europe. Data themselves, however, are not part of ricu and while the supported datasets are publicly available, access to each of the data sources has to be requested separately. Four of the datasets—namely, MIMIC-III, MIMIC-IV, eICU, and HiRID—are hosted on PhysioNet [22], while the fifth, AmsterdamUMCdb, is currently distributed via a separate platform [23].

For the MIMIC-III and eICU datasets, small subsets of data are available as demo datasets that do not require credentialed access to PhysioNet. As the terms for distribution of these demo datasets are less restrictive, they can be made available as data packages mimic.demo and eicu.demo. Due to size constraints, however, they are not available via CRAN but can be installed from GitHub as:

install.packages(

c("mimic.demo", "eicu.demo"),

repos = "https://eth-mds.github.io/physionet-demo"

)

The demo datasets are especially handy for users who wish to investigate the capabilities of ricu. Demo datasets can be set up in a matter of minutes, and the user can explore much of the functionality offered by ricu using just the demo data.

The recommended way of setting up the full data, after data access is granted, is the following. Credentials can either be provided as environment variables (RICU_PHYSIONET_USER and RICU_PHYSIONET_PASS for access to PhysioNet data, as well as RICU_AUMC_TOKEN for AmsterdamUMCdb) and if the corresponding variables are unset, user input is again required in interactive sessions. For noninteractive sessions, functionality is exported such that data can be downloaded and set up ahead of first access (see ?setup_src_data for the documentation).

Contingent on being granted access by the data owners, download requires a stable Internet connection, as well as 50 to 100 GB of temporary disk storage for unpacking and preparing the data for efficient access. In terms of permanent storage, 5 to 10 GB per dataset are required (see Table 1), while memory requirements are kept reasonably low by iterating over row-chunks for setup operations. Laptop class hardware (8–16 GB of memory) should suffice for setup and many analysis tasks that focus only on subsets of rows (and columns). Initial data source setup (depending on available download speeds and CPU/disk type) may take upward of an hour per dataset. In Table 1, we provide a summary of the available datasets, giving the user some idea about the different data sources. A more detailed discussion of the data sources, and in particular how they are represented within ricu, is given in Supplementary Material B. For other data source inquiries, we refer the user to the original documentation of the datasets or to the recent review paper that analyzes some of upsides and downsides of each of them [24].

**Table 1:** Comparison of datasets supported by ricu, highlighting some of the major similarities and distinguishing features. Values followed by parenthesized ranges represent medians and are accompanied by interquartile ranges.

	MIMIC-III	eICU	AmsterdamUMCdb	HiRID	MIMIC-IV
Number of tables	26	31	7	5	27
Disk storage (GB)	6.04	6.50	10.81	4.52	10.33
Largest table (rows)	330,712,483	151,604,232	977,625,612	776,921,131	329,499,788
Available concepts*	89	87	85	74	87
**Data collection**					
Time span	2001–2012	2014–2015	2003–2016	2008–2016	2008–2019
Country of origin	United States	United States	Netherlands	Switzerland	United States
**Admission counts**					
ICU	61,532	200,859	23,106	33,904	76,540
Hospital	57,841	166,355	–	–	69,300
Unique patients	46,476	–	20,109	–	53,150
**Stay lengths (day)**					
ICU stays	2.09 (1.11–4.48)	1.57 (0.82–2.97)	1.07 (0.84–3.67)	0.99 (0.81–2.16)	1.93 (1.09–3.73)
Hospital stays	6.57 (3.80–11.86)	5.05 (2.71–9.03)	–	–	6.62 (3.87–11.36)
**Vital signs (1/hour)**					
Heart rate	1.00 (1.00–1.02)	12.00 (12.00–12.00)	60.00 (60.00–60.00)	30.00 (30.00–60.00)	1.00 (1.00–1.00)
Mean arterial pressure	1.00 (1.00–1.33)	12.00 (4.00–12.00)	60.00 (60.00–60.00)	30.00 (30.00–60.00)	1.00 (1.00–1.02)
**Lab tests (1/day)**					
Bilirubin	1.00 (0.86–1.38)	1.00 (0.91–1.20)	1.00 (0.33–1.06)	1.00 (0.98–1.04)	1.00 (0.94–1.30)
Lactate	4.72 (1.84–10.75)	3.78 (1.54–6.67)	7.42 (4.30–14.12)	4.66 (2.96–7.96)	4.68 (2.29–9.47)

* These values represent the number of atomic concepts per data source. Additionally, 27 recursive concepts are available, which build on data source–specific atomic concepts in a source-agnostic manner (see Supplementary Material C for details).

### Adding external datasets

As mentioned earlier, ricu is designed with extensibility in mind, also allowing the user to add their own data, including data that are not publicly available. However, the addition of custom datasets requires several configuration steps, which are outlined in the “Adding external datasets” section of Supplementary Material D.

### Concepts

Concepts are the main building blocks of the ricu package. Most of the concepts that are ready-to-use with ricu are grounded in the OMOP Vocabulary, but not all, since some are not covered by the vocabulary. Concepts can be loaded using the integer OMOP concept ID or the abbreviated concept name strings, for fostering efficiency of code writing. Currently, there are 119 concepts available in ricu, which fall into 4 broad groups. We refer to this set of concepts as the ricu  *dictionary*. In Table 2, we provide an overview of the groups, categories within each group, and the number of available concepts in each category. Data on any of the concepts can be loaded using the load_concepts() function, which is the main workhorse of the package. The function outputs 3 possible data types, which are the following:

- Data with no time stamps (such as the concept sex, OMOP concept ID 37116947) output the type id_tbl, for example:
# An `id_tbl`: 3 x 2
# Id var:   `icustay_id`
 icustay_id omop_37116947
    <int> <chr>
1   201006 Male
2   201204 Female
3   203766 Female
  This output format has only the patient identifier and the value of the concept.
- Data with time stamps (such as lactate levels, OMOP concept ID 4191725, but can also be loaded using the abbreviation "lact" with the result shown below) output the type ts_tbl, formatted as:
# A `ts_tbl`: 3 x 3
# Id var:   `icustay_id`
# Units:   `lact` [mmol/L]
# Index var: `charttime` (1 hours)
 icustay_id charttime lact
    <int> <drtn>  <dbl>
1   201006 -58 hours  1.7
2   201006 -10 hours  1.8
3   201006  0 hours  2.2
  This output format has the patient identifier, the time stamp, and the value of the concept.
- Data with start and end time stamps (such as mechanical ventilation) output the type win_tbl:
# A `win_tbl`: 3 x 4
# Id var:    `admissionid`
# Index var:  `start` (1 hours)
# Duration var: `dur_var`
 admissionid start  dur_var mech_vent
    <int> <drtn> <drtn>  <chr>
1      0 5 hours 810 mins invasive
2      1 0 hours 405 mins invasive
3      2 0 hours 159 mins invasive
  This output format has the patient identifier, the time stamp of the start point, the duration of the concept, and the value of the concept.

Using the load_concepts() function, the user can also load multiple concepts at the same time. In that case, one of the above data types is returned. If the data are queried using OMOP concept IDs, the column names of the resulting output will contain the corresponding IDs, whereas if abbreviated string names are used, the output will have these as column names. For a more detailed explanation of the output returned by load_concepts() in case of loading multiple concepts, we refer the reader to Supplementary Material C.

**Table 2:** Number of currently available concepts in ricu grouped by category

Group	Category	Count
Physiology	Blood gas	10
	Chemistry	21
	Hematology	20
	Neurological	7
	Output	2
	Respiratory	10
	Vitals	6
Treatment	Medications	17
	Microbiology	1
Demographics	Demographics	6
Outcomes	Outcome	19

### Adding concepts

When interested in using a concept, the user can first try to investigate whether the concept is available within ricu. If this is not the case, ricu provides a mechanism for extending the dictionary with arbitrary user-specified concepts. The recommended way of specifying a concept is using a JSON-formatted text file. As an illustration, we show how the concept of heart rate (which already exists in the dictionary) could be specified by the user, if it were not available. The following entry would create a concept hr for the MIMIC-III demo dataset:

{

"hr": {

"unit": ["bpm", "/min"],

"min": 0,

"max": 300,

"omopid": 4239408,

"description": "heart rate",

"category": "routine vital signs",

"sources": {

"mimic_demo": [

{

"ids": [211, 220045],

"table": "chartevents",

"sub_var": "itemid"

}

]

}

}

}

After creating such a JSON-formatted text file, the user needs to set the environment variable RICU_CONFIG_PATH to the folder where the file is located. Upon doing so, the user can use the load_concepts() function to load the concept hr. The heart rate values would be loaded from the chartevents table in MIMIC-III, by taking the subset of rows for which the value in the column itemid equals either 221 or 220045, which are the codes corresponding to heart rate. Values lower than 0 and higher than 300 would be removed from the returned result (the max and min fields are not mandatory in general). An alternative method for specifying a concept would be to use the concept() and item() functionality. This alternative method is illustrated in a worked example in the Results section.

In general, especially when preprocessing is required, specifying a concept can be more involved. In this case, the user can input a *callback* function that performs arbitrary preprocessing on the data that are loaded, before it is returned by load_concepts(). For more details, we refer the reader to Supplementary Material C, where concept loading and specification are explained in more depth.

### Other functionality

Beyond the data download and setup functionality, as well as the load_concepts() function, the ricu package also has a number of helper functions for working with the data. For example, there is functionality for handling time-series data, taking minima or maxima over time windows, imputing values, and many more. In the interest of space, we do not go into full detail about this additional supporting functionality but rather refer the user to the package documentation.

## Results

The capabilities of the ricu package are perhaps best illustrated by using it for 2 case studies. We apply ricu to first study the association of lactate and mortality and then study the association of insulin dosage and diabetes. The 2 examples are intended to showcase specific aspects of the package discussed in the Findings section.

### Lactate and mortality

First, the association of lactate levels and mortality is investigated. This problem has been studied in the literature, and it is widely accepted that both static and dynamic lactate indices are associated with increased mortality [25–27]. In order to understand the relationship of lactate and mortality, we fitted a time-varying proportional hazards Cox model [28, 29] to the time-series data which includes the Sequential Organ Failure Assessment (SOFA) score (as a general predictor of illness severity) and the lactate values. The analysis was performed on the MIMIC-III demo data. Furthermore, for the sake of this example, the patient cohort consists of patients between 20 and 90 years of age.

R> src <- "mimic_demo"

R>

R> cohort <- load_concepts("age", src)

R>

R> dat <- load_concepts(

+  c("lact", "death", "sofa"), src,

+  patient_ids = cohort [age > 20 &  age < 90, ]

+  )

R>

R> dat <- dat[,

+  head(.SD, n = match(TRUE, death, .N)),

+  by = c(id_vars(dat))

+ ]

R>

R> dat <- fill_gaps(dat)

R>

R> dat <- replace_na(

+  dat, c(NA, FALSE),

+  type = c("locf", "const"),

+  vars = c("lact", "death"),

+  by = id_vars(dat), by_ref = TRUE)

+ )

R>

R> cox_mod <- coxph(

+  Surv(charttime - 1L, chart time, death) ~

+   lact + sofa,

+  data = dat

+ )

**Figure 1:**
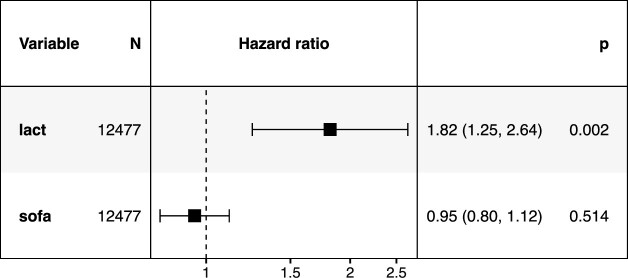
Forest plot for the hazard ratio of lactate levels obtained on the MIMIC-III demo dataset.

After loading the data, some minor preprocessing is still required before modeling: first, data are filtered such that only data up to (and including) the hour in which the death flag switches to TRUE are used. After this, missing values for lact are imputed using a last observation carry forward (LOCF) scheme (observing the patient grouping), and missing death values are set to FALSE (contained in the usage of the replace_na() function). The resulting fit of the proportional hazards model can be visualized in Fig. 1.

A simple exploration already shows that the increased values of lactate are associated with mortality, even after adjusting for the SOFA score. Using the abstraction provided by ricu, this analysis can also be applied to other datasets with minimal effort by simply changing the src argument and rerunning the code above.

### Diabetes and insulin treatment

For the next example, now using the full MIMIC-III data, we illustrate the usage of comorbidities and treatment-related information. First, we focus on the amount of insulin administered to patients in the first 24 hours from their ICU admission. As the ricu dictionary already contains an insulin concept called ins, we introduce the ins24 concept, which builds on top of the existing ins and represents the total amount of insulin administered in first 24 hours of the ICU stay. This can be implemented by specifying the ins24 concept as a *recursive concept* (rec_cncpt), requesting data from ins.

In order to calculate the total amount of administered insulin, it is required to change the default aggregation method from median() to sum(). Failing to do so would yield underreported values whenever several insulin administrations fall within a given time step. The callback function ins_cb() shown below is then inserted into the loading process, performing the preprocessing steps outlined above: first, data are subsetted to fall into the first 24 hours of ICU admissions, followed by binning of summed values.

R> ins_breaks <- c(0, 1, 10, 20, 40, Inf)

R>

R> ins_cb <- function(ins, ...) {

+

+ day_one <- function(x) x >= hours(0L) &

+            x <= hours(24L)

+  idx_var <- index_var(ins)

+  ids_var <- id_vars(ins)

+

+  ins <- ins[

+   day_one(get(idx_var)), list(ins24 = sum(ins)),

+   by = c(ids_var)

+  ]

+  ins <- ins[,

+   ins24 := list(cut(ins24, breaks = ins_breaks,

+            right = FALSE))

+  ]

+  ins

+ }

The ins24 concept can then be specified by adding the following entry into the JSON-formatted configuration file specifying additional concepts:

{

"ins24": {

"concepts": "ins",

"description": "Insulin in first 24 hours",

"callback": "ins_cb",

"class": "rec_cncpt",

"aggregate": "sum",

"target": "id_tbl"

}

}

which then makes the concept ins24 available for the load_concepts() function. Next, we want to obtain the diabetic status of the patients in the database. The diabetes concept can be implemented as lgl_cncpt (as diabetes is a binary variable) by matching International Classification of Diseases, Ninth Revision (ICD-9) codes using a regular expression. For creating the required callback function, which produces a logical vector, the exported function factory transform_fun() can be employed, coupled with a function like grep_diab() (matching all ICD-9 codes starting with 250), performing the desired transformation.

R> grep_diab <- function(x) {

+  grepl("^250\\.?[0-9]{2}$", x)

+ }

To specify the diabetes concept diab into the dictionary, the following entry is added to the configuration file:

{

"diab": {

"class": "lgl_cncpt",

"description": "diabetes status",

"target": "id_tbl",

"sources": {

"mimic": [

{

"table": "diagnoses_icd",

"class": "col_itm",

"callback": "transform_fun(grep_diab)"

}

]

}

}

}

Finally, with both the ins24 and diab concepts ready, we can perform an analysis of the association of diabetes with insulin administration, using the following code:

R> src <- "mimic"

R> diab <- item(

+ src, table = "diagnoses_icd",

+  callback = transform_fun(grep_diab),

+  class = "col_itm"

+ )

R>

R> diab <- concept("diab", diab, "diabetes",

+         target = "id_tbl",

+         class = "lgl_cncpt")

R>

R> dat <- load_concepts(c("ins24", "diab"), src,

+            id_type = "icustay")

R> dat <- replace_na(dat, "[0,1)", vars = "ins24")

**Figure 2:**
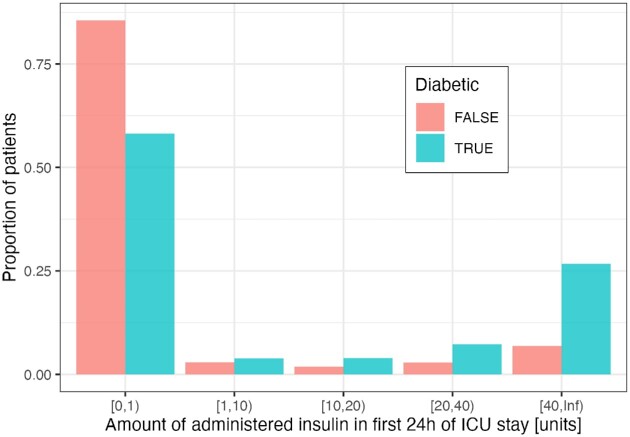
Association of diabetes and the amount of insulin administered in the first 24 hours of the ICU stay in the MIMIC-III dataset. The difference between the diabetic and nondiabetic groups is statistically significant for each insulin bin (*P* < 0.001 for each χ^2^ test).

Following this, the difference between the 2 groups can be visualized with a histogram over the binned insulin administration values, as shown in Fig. 2. The plot suggests that during the first day of the ICU stay, perhaps unsurprisingly, diabetic patients are more likely to receive a large amount of administered insulin (*P* < 0.001 using a χ^2^ test for the null hypothesis that diabetes and insulin amount are independent).

## Discussion and conclusion

We developed the ricu R-package for handling 5 large publicly available ICU datasets. It allows in its current form the user to load various clinical concepts through a unified interface, abstracting out dataset specifics and shielding the user from the data-cleaning and data-mining process. The available concepts include physiological, demographic, and treatment-related information and also some important clinical outcomes. Most of the concepts in the package are grounded in the OMOP Vocabulary, although we found that this is a nontrivial effort in practice due to the lack of documentation across different data sources and the heterogeneous nature of the different datasets. Importantly, the package we developed was designed with extensibility in mind. In particular, the user can extend the existing concept dictionary of the package and also add external data sources, which can then be analyzed in combination with the existing publicly available data.

The main implication of our work is that a software tool is now available that aims to shield researchers from developing computational infrastructure completely anew. In particular, the extensible design implies that researchers can leverage the infrastructure of the ricu package even if their question of interest requires clinical concepts that are beyond the current scope of the package. Furthermore, researchers using their own data now have an opportunity to import their data into the ricu package and analyze the data in conjunction with the publicly available data, thereby allowing them to test the external validity of their scientific findings. Another implication of our work is that developing guidelines for publicly sharing ICU data would be a worthwhile undertaking. Currently, the publicly available datasets are very heterogeneous, both in terms of design and available documentation, and rarely provide explicit mappings to existing vocabularies/ontologies, such as the one used by OMOP.

To the best of our knowledge, there are no previous software tools that serve the same purpose as the ricu package. The most similar work to ours, in which the authors aim to abstract away some of the dataset specifics, has so far exclusively focused on the MIMIC dataset [19]. Therefore, we hope that the ricu package can become a community-wide effort that enables additions or modifications of new or existing concepts. Furthermore, ricu easily allows for the addition of new datasets, and we therefore expect the number of available data sources to grow over time.

The main strength of the ricu package is the ease with which it allows the user to set up and analyze multiple datasets through a unified interface. Furthermore, the breadth of the datasets supported by the package, which originate from 3 countries on 2 different continents, is another strength of our work. This allows users to test their hypothesis in different populations, making their findings more likely to be biologically plausible and relevant. One current limitation of the ricu package is that the dictionary available with the package is not comprehensive. However, we expect to develop the package further and hope that the ricu dictionary will grow over time. One final limitation we mention is that not every concept in the ricu dictionary is mapped to the OMOP Vocabulary, since some of the concepts do not exist therein. Therefore, mapping them onto the ontology is left for future work, once they become available within it.

In conclusion, the ricu package, developed in this article, allows the user to load 119 clinical concepts from the MIMIC-III, MIMIC-IV, eICU, AUMCdb, and HiRID datasets. The package can now be used by the research community to save time by circumventing the process of developing computational infrastructure, to foster reproducible research, and to allow researchers to test the external validity and robustness of their scientific hypotheses and models.

## Availability of source code

- Project name: ricu
- Project home page: https://github.com/eth-mds/ricu
- RRID: SCR_023318
- Biotools: ricu
- Operating system(s): Platform independent
- Programming language: R
- Other requirements: —
- License: GNU GPL3

## Supplementary Material

giad041_GIGA-D-22-00339_Original_Submission

giad041_GIGA-D-22-00339_Revision_1

giad041_GIGA-D-22-00339_Revision_2

giad041_GIGA-D-22-00339_Revision_3

giad041_GIGA-D-22-00339_Revision_4

giad041_Reviewer_1_Report_Original_Submission Chris Armit -- 2/7/2023 Reviewed

giad041_Reviewer_2_Report_Original_Submission Emre Guney, Ph.D. -- 3/1/2023 Reviewed

giad041_Reviewer_2_Report_Revision_1 Emre Guney, Ph.D. -- 3/9/2023 Reviewed

giad041_Reviewer_2_Report_Revision_2 Emre Guney, Ph.D. -- 4/14/2023 Reviewed

giad041_Reviewer_2_Report_Revision_3 Emre Guney, Ph.D. -- 5/2/2023 Reviewed

giad041_Supplemental_Files

## Data Availability

All of the data handled by the ricu package are publicly available. The MIMIC-III and eICU demo datasets can be accessed immediately from GitHub by running remotes::install_github("eth-mds/eicu-demo"). For the full datasets, access requests are needed to respective dataset owners. Four of the 5 datasets (MIMIC-III, MIMIC-IV, eICU, and HiRID) are hosted on PhysioNet [22], while the fifth, AmsterdamUMCdb, is currently distributed via a separate platform [23]. Snapshots of our code and other data further supporting this work are openly available in the *GigaScience* repository, GigaDB [30].

## Additional files

**Supplementary Material A** - Quick Start

**Supplementary Material B** - Data Sources

**Supplementary Material C** - Data Concepts

**Supplementary Material D** - Implementation Details

**Supplementary Fig. S1**. Making a dataset available to ricu involves several steps, starting with data download, followed by preparation for efficient access and finalized by instantiation of data structures containing relevant metadata. The functions that are used for each step are displayed above arrows and below (in red) are indicated specific configuration settings or environment variables that are needed for (or can be used to customize) the specific step.

**Supplementary Fig. S2**. Data loading proceeds through several layers, each contributing a step toward harmonizing discrepancies among raw data representations provided by the different data sources. Raw data tables are represented by ricu as src_tbl objects, which can be queried using load_src(). Absolute time stamps in the returned data.table are converted to times relative to admission (in minutes) by load_difftime(), and finally, load_id()/load_ts()/load_win() ensures a given ID system and time interval.

## Abbreviations

AmsterdamUMCdb: Amsterdam University Medical Center Database; BIDMC: Beth Israel Deaconess Medical Center; CRAN: Comprehensive R Archive Network; eICU: electronic intensive care unit; HiRID: High Time-Resolution ICU Dataset; ICD-9: International Classification of Diseases, Ninth Revision; ICU: intensive care unit; ID: identifier; ML: machine learning; OMOP: Observational Medical Outcomes Partnership; SOFA: Sequential Organ Failure Assessment.

## References

[1] Evans RS. Electronic health records: then, now, and in the future. Yearb Med Inform. 2016;25(Suppl. 1):48–61. 10.15265/IYS-2016-s006 PMC5171496 27199197

[2] Jiang F, Jiang Y, Zhi H et al. Artificial intelligence in healthcare: past, present and future. Stroke Vasc Neurol. 2017;2(4):230–43. 29507784 10.1136/svn-2017-000101 PMC5829945

[3] Pickering BW, Gajic O, Ahmed A, et al. Data utilization for medical decision making at the time of patient admission to ICU. Crit Care Med. 2013;41(6):1502–10. 23528804 10.1097/CCM.0b013e318287f0c0

[4] Desautels T, Calvert J, Hoffman J et al. Prediction of sepsis in the intensive care unit with minimal electronic health record data: a machine learning approach. JMIR Med Inform. 2016;4(3):e28. 27694098 10.2196/medinform.5909 PMC5065680

[5] Nemati S, Holder A, Razmi F et al. An interpretable machine learning model for accurate prediction of sepsis in the ICU. Crit Care Med. 2018;46(4):547–53. 29286945 10.1097/CCM.0000000000002936 PMC5851825

[6] Futoma J, Hariharan S, Heller K, et al. An improved multi-output Gaussian process RNN with real-time validation for early sepsis detection. In: 2017 Machine Learning for Healthcare Conference, PMLR, 2017. (pp. 243–254).

[7] Kam HJ, Kim HY. Learning representations for the early detection of sepsis with deep neural networks. Comput Biol Med. 2017;89:248–55. 28843829 10.1016/j.compbiomed.2017.08.015

[8] Fleuren LM, Klausch TLT, Zwager CL, et al. Machine learning for the prediction of sepsis: asystematic review and meta-analysis of diagnostic test accuracy. Intensive Care Med. 2020;46(3):383–400. 31965266 10.1007/s00134-019-05872-y PMC7067741

[9] Johnson AE, Pollard TJ, Shen L, et al. MIMIC-III, a freely accessible critical care database. Sci Data. 2016;3:160035. 27219127 10.1038/sdata.2016.35 PMC4878278

[10] Wong A, Otles E, Donnelly JP et al. External validation of a widely implemented proprietary sepsis prediction model in hospitalized patients. JAMA Intern Med. 2021;181(8):1065–1070. 34152373 10.1001/jamainternmed.2021.2626 PMC8218233

[11] Singer M, Deutschman CS, Seymour CW et al. The Third International Consensus Definitions for Sepsis and Septic Shock (Sepsis-3). JAMA. 2016;315(8):801–10. 26903338 10.1001/jama.2016.0287 PMC4968574

[12] Wang RZ, Sun CH, Schroeder PH et al. Predictive models of sepsis in adult ICU patients. In: 2018 IEEE International Conference on Healthcare Informatics (ICHI) Institute of Electrical and Electronics Engineers. IEEE, 2018. p. 390–1.

[13] Johnson AEW, Aboab J, Raffa JD et al. A comparative analysis of sepsis identification methods in an electronic database. Crit Care Med. 2018;46(4):494–9. 29303796 10.1097/CCM.0000000000002965 PMC5851804

[14] Pollard TJ, Johnson AE, Raffa JD et al. The eICU collaborative research database, a freely available multi-center database for critical care research. Sci Data. 2018;5:180178. 30204154 10.1038/sdata.2018.178 PMC6132188

[15] Faltys M, Zimmermann M, Lyu X et al. HiRID, a high time-resolution ICU dataset (version 1.1.1). 2021. PhysioNet. https://physionet.org/content/hirid/1.1.1/. Accessed 25 April 2022.

[16] Thoral PJ, Peppink JM, Driessen RH et al. Sharing ICU patient data responsibly under the Society of Critical Care Medicine/European Society of Intensive Care Medicine Joint Data Science Collaboration: The Amsterdam University Medical Centers Database (AmsterdamUMCdb) example. Crit Care Med. 2021;49 (6):e563 33625129 10.1097/CCM.0000000000004916 PMC8132908

[17] Johnson A, Bulgarelli L, Pollard T, et al. MIMIC-IV (Version 1.0). 2021. https://physionet.org/content/mimiciv/1.0/. Accessed 25 April 2022. PhysioNet.

[18] Adibuzzaman M, Musselman K, Johnson A et al. Closing the data loop: an integrated open access analysis platform for the MIMIC database. In: 2016 Computing in Cardiology Conference (CinC) Institute of Electrical and Electronics Engineers. IEEE, 2016. p. 137–40. 10.23919/CIC.2016.7868698 PMC5630151 28989936

[19] Wang S, McDermott MB, Chauhan G et al. MIMIC-Extract: a data extraction, preprocessing, and representation pipeline for MIMIC-III. In: Proceedings of the ACM Conference on Health, Inference, and Learning Association for Computing Machinery. IEEE, 2020. p. 222–35.

[20] Glicksberg BS, Oskotsky B, Giangreco N et al. ROMOP: a light-weight R package for interfacing with OMOP-formatted electronic health record data. JAMIA Open. 2019;2(1):10–14. 31633087 10.1093/jamiaopen/ooy059 PMC6800657

[21] Glicksberg BS, Oskotsky B, Thangaraj PM, et al. PatientExploreR: an extensible application for dynamic visualization of patient clinical history from electronic health records in the OMOP common data model. Bioinformatics. 2019;35(21):4515–8. 31214700 10.1093/bioinformatics/btz409 PMC6821222

[22] Goldberger AL, Amaral LAN, Glass L, et al. PhysioBank, PhysioToolkit and PhysioNet. Circulation. 2000;101(23): e215–20. 10851218 10.1161/01.cir.101.23.e215

[23] Amsterdam University Medical Center’s Database Collaborators and the SCCM/ESICM Joint Data Science Task Force, Amsterdam University Medical Center Database . 2020. https://amsterdammedicaldatascience.nl/amsterdamumcdb/. Accessed 18 May 2023.

[24] Sauer CM, Dam TA, Celi LA et al. Systematic review and comparison of publicly available ICU data sets—a decision guide for clinicians and data scientists. Crit Care Med. 2022;50(6):e581–8. 35234175 10.1097/CCM.0000000000005517 PMC9150442

[25] Haas SA, Lange T, Saugel B et al. Severe hyperlactatemia, lactate clearance and mortality in unselected critically ill patients. Intensive Care Med. 2016;42(2):202–10. 26556617 10.1007/s00134-015-4127-0

[26] Nichol A, Bailey M, Egi M et al. Dynamic lactate indices as predictors of outcome in critically ill patients. Crit Care. 2011;15(5):R242. 22014216 10.1186/cc10497 PMC3334793

[27] Van Beest PA, Brander L, Jansen SP et al. Cumulative lactate and hospital mortality in ICU patients. Ann Intensive Care. 2013;3(1):6. 23446002 10.1186/2110-5820-3-6 PMC3599274

[28] Therneau TM, Grambsch PM. Modeling Survival Data: Extending the Cox Model. New York: Springer-Verlag, 2000. 10.1007/978-1-4757-3294-8.

[29] Therneau TM. A Package for Survival Analysis in R. 2021.; https://CRAN.R-project.org/package=survival. Accessed 18 May 2023.

[30] Nicolas B, Drago P, Ida-Fong U et al. Supporting data for “ricu: R’s Interface to Intensive Care Data.” GigaScience Database. 2023. 10.5524/102392.

