# Supplementary material for: ricu: R’s interface to intensive care data
Source: Gigascience. 2023 Jun 15;12:giad041. doi: 10.1093/gigascience/giad041 (PMC10268223; doi:10.1093/gigascience/giad041)
Supplement: giad041_GIGA-D-22-00339_Revision_4 [file giad041_giga-d-22-00339_revision_4.pdf]

GigaScience
ricu: R's Interface to Intensive Care Data
--Manuscript Draft--

**Manuscript Number:**	GIGA-D-22-00339R4
**Full Title:**	ricu: R's Interface to Intensive Care Data
**Article Type:**	Technical Note
**Funding Information:**	Personalized Health and Related Technologies (#2017-110) — Dr. Drago Plecko, Dr. Nicolas Bennett
**Abstract:**	Objective: To develop a unified framework for analyzing data from five large, publicly available ICU datasets.<br>Findings: Using three American (MIMIC-III, MIMIC-IV, eICU), and two European (AUMCdb, HiRID) databases, we constructed a mapping for each database to a set of clinically relevant concepts, and grounded these concepts in an existing ontology wherever possible (in particular, we used SNOMED CT). Furthermore, we performed synchronization in the units of measurement, and data type representation. On top of this, we built functionality which allows the user to download, set up, and load data from all of the five databases, through a unified Application Programming Interface (API). The resulting \pkg{ricu} R-package represents the computational infrastructure for handling publicly available ICU datasets and its latest release allows the user to load 119 existing clinical concepts from the five data sources.<br>Conclusion: The ricu R-package (available on Github and CRAN) is the first tool that enables users to analyze publicly available ICU datasets simultaneously (datasets are available upon request from respective owners). Such an interface saves researchers time when analyzing ICU data, and helps reproducibility. We hope that ricu can become a community-wide effort, so that data harmonization is not repeated by each research group separately. One current limitation is that concepts were added on a case-to-case basis, and therefore the resulting dictionary of concepts is not comprehensive. Further work is needed to make the dictionary comprehensive.
**Corresponding Author:**	Drago Plecko<br>ETH Zürich: Eidgenossische Technische Hochschule Zurich<br>SWITZERLAND
**Corresponding Author Secondary Information:**	
**Corresponding Author's Institution:**	ETH Zürich: Eidgenossische Technische Hochschule Zurich
**Corresponding Author's Secondary Institution:**	
**First Author:**	Drago Plecko
**First Author Secondary Information:**	
**Order of Authors:**	Drago Plecko
	Nicolas Bennett
	Ida-Fong Ukor
	Nicolai Meinshausen
	Peter Bühlmann
**Order of Authors Secondary Information:**	
**Response to Reviewers:**	The authors would like to thank the reviewers and editors for the constructive and timely review process.<br><br>Please find enclosed an updated version of the manuscript. The authors hope they understood the formatting instructions correctly.

Powered by Editorial Manager® and ProduXion Manager® from Aries Systems Corporation

	Best wishes,<br>ricu authors
**Additional Information:**	
**Question**	**Response**
Are you submitting this manuscript to a special series or article collection?	No
**Experimental design and statistics**<br><br>Full details of the experimental design and statistical methods used should be given in the Methods section, as detailed in our Minimum Standards Reporting Checklist. Information essential to interpreting the data presented should be made available in the figure legends.<br><br>Have you included all the information requested in your manuscript?	Yes
**Resources**<br><br>A description of all resources used, including antibodies, cell lines, animals and software tools, with enough information to allow them to be uniquely identified, should be included in the Methods section. Authors are strongly encouraged to cite Research Resource Identifiers (RRIDs) for antibodies, model organisms and tools, where possible.<br><br>Have you included the information requested as detailed in our Minimum Standards Reporting Checklist?	Yes
**Availability of data and materials**<br><br>All datasets and code on which the conclusions of the paper rely must be either included in your submission or deposited in publicly available repositories (where available and ethically appropriate), referencing such data using a unique identifier in the references and in the “Availability of Data and Materials”	Yes

section of your manuscript.<br><br>Have you have met the above requirement as detailed in our Minimum Standards Reporting Checklist?	

Powered by Editorial Manager® and ProduXion Manager® from Aries Systems Corporation

Manuscript
Click here to access/download;Manuscript;main.tex

GigaScience, 2023, 1–8

doi: xx.xxxx/xxxx
Manuscript in Preparation
Technical Note

TECHNICAL NOTE

ricu: R's Interface to Intensive Care Data

Nicolas Bennett[1,†], Drago Plečko[1,†,], Ida-Fong Ukor[2], Nicolai Meinshausen[1] and Peter Bühlmann[1]

[1]Seminar for Statistics, ETH Zürich and [2]Department of Anaesthesiology and Perioperative Medicine, Monash Health

*Correspondence email address: drago.plecko@stat.math.ethz.ch
†Contributed equally.

Abstract

Objective: To develop a unified framework for analyzing data from five large, publicly available ICU datasets.
Findings: Using three American (MIMIC-III, MIMIC-IV, eICU), and two European (AUMCdb, HiRID) databases, we constructed a mapping for each database to a set of clinically relevant concepts, which are grounded in the OMOP Vocabulary wherever possible. Furthermore, we performed synchronization in the units of measurement, and data type representation. On top of this, we built functionality which allows the user to download, set up, and load data from all of the five databases, through a unified Application Programming Interface (API). The resulting **ricu** R-package represents the computational infrastructure for handling publicly available ICU datasets and its latest release allows the user to load 119 existing clinical concepts from the five data sources.
Conclusion: The **ricu** R-package (available on Github and CRAN) is the first tool that enables users to analyze publicly available ICU datasets simultaneously (datasets are available upon request from respective owners). Such an interface saves researchers time when analyzing ICU data, and helps reproducibility. We hope that **ricu** can become a community-wide effort, so that data harmonization is not repeated by each research group separately. One current limitation is that concepts were added on a case-to-case basis, and therefore the resulting dictionary of concepts is not comprehensive. Further work is needed to make the dictionary comprehensive.

Keywords: Intensive Care Medicine; Electronic Health Records; Computational Methods

Introduction

Collection of electronic health records has seen a significant rise in recent years [1], opening up opportunities and providing the grounds for a large body of data-driven research oriented towards helping clinicians in decision-making and therefore improving patient care and health outcomes [2]. While growing amounts of collected patient data might not be easily utilized by intensivists for decision-making [3], this poses an opportunity for the application of machine learning (ML) methods.

One example of a problem that has received much attention from the ML community is early prediction of sepsis in the intensive care unit (ICU) [4, 5, 6, 7]. Interestingly, there is evidence that a large proportion of the publications on this topic are based on the same dataset [8], the Medical Information Mart for Intensive Care III (MIMIC-III) [9], showing a systematic lack of external validation. This issue has recently again been highlighted by a study demonstrating poor performance in external validation of a widely adopted sepsis prediction model [10].

Contributing to this problem might well be the lack of computational infrastructure for handling multiple datasets. The MIMIC-III dataset consists of 26 different tables containing about 20GB of data. While much work and care has gone into data pre-processing in order to provide a self-contained ready-to-use data resource with MIMIC-III, seemingly simple tasks such as computing a Sepsis-3 label [11] remain non-trivial efforts[1]. This is only exacerbated when aiming to co-integrate multiple different datasets of this form, spanning hospitals and even countries, in order to capture effects of differing practice and demographics.

Given the somewhat specific focus of ICU data, it may come

1 There is considerable heterogeneity in the number of patients satisfying the Sepsis-3 criterion [11] among studies investigating MIMIC-III. Reported Sepsis-3 prevalence ranges from 11.3% [4], over 23.9% [5] and 25.4% [12], up to 49.1% [13]. While some of this variation may be explained by differing patient inclusion criteria, differences in label implementation must also contribute significantly.

Compiled on: May 18, 2023.
Draft manuscript prepared by the author.

Key Points

- The **ricu** R-package is the first tool that enables users to analyze five large ICU datasets through a unified interface,
- **ricu** currently supports 119 clinical concepts across MIMIC-III, MIMIC-IV, eICU, HiRID, and AUMCdb datasets,
- **ricu** allows for easy addition of user-specified concepts and datasets, integrating them with the existing infrastructure.

as a surprise as to how heterogeneous the currently available datasets are. In MIMIC-III and HiRID, for example, time-stamps are reported as absolute times (albeit randomly shifted due to data privacy concerns), whereas eICU and AmsterdamUMCdb use relative times (with origins being admission times). Another example involves different types of patient identifiers and their use among datasets. Common to all is the notion of an ICU admission identifier (ID), but apart from that, the amount of available information varies: While ICU (and hospital) readmissions for a given patient can be identified in some, this is not possible in other datasets. Furthermore, use of identifier systems might not be consistent over tables. In MIMIC-III, for example, some tables refer to ICU stay IDs while others use hospital stay IDs, which slightly complicates data retrieval for a fixed ID system. Additionally, table layouts vary (*long* versus *wide* data arrangement) and data organization in general is far from consistent over datasets.

In light of the above described background, the aim of **ricu** is to provide computational infrastructure allowing users to investigate complex research questions in the context of critical care medicine as easily as possible, by introducing a unified interface to a heterogeneous set of data sources. The package enables users to write dataset-agnostic code which can simplify implementation and shorten the time necessary for prototyping code querying different datasets. In its current form, the package handles five large-scale, publicly available intensive care databases out of the box: MIMIC-III [9] from the Beth Israel Deaconess Medical Center (BIDMC) in Boston, Massachusetts, the eICU Collaborative Research Database [14], containing data collected from 208 hospitals across the United States, the High Time Resolution ICU Dataset (HiRID) [15] from the Department of Intensive Care Medicine of the Bern University Hospital, Switzerland, the Amsterdam University Medical Center Database (AmsterdamUMCdb) [16] from the Amsterdam University Medical Center, and MIMIC-IV [17], again using data from BIDMC. Furthermore, **ricu** was designed with extensibility in mind such that adding new public and/or private user-provided datasets is possible. Being implemented in R, a programming language popular among statisticians and data analysts, it is our hope to contribute to accessible and reproducible research by using a familiar environment and requiring only few system dependencies, thereby considerably simplifying setup.

To our knowledge, infrastructure that provides a common interface to multiple ICU datasets is a novel contribution. While there have been efforts [18, 19] attempting to abstract away some specifics of a dataset, these have so far exclusively focused on MIMIC-III, the most popular of public ICU datasets, and have not been designed with dataset interoperability in mind. It is also worth mentioning software packages **ROMOP** [20] and **PatientExploreR** [21] that offer some useful infrastructure for data compatible with the Observational Medical Outcomes Partnership (OMOP) common data model, but the heterogeneity of the datasets supported by **ricu** limits the ability to organize them according to such a data model.

Findings

The **ricu** package can be accessed from either Github or CRAN, and the lastest development version can be installed by running:

```
devtools::install_github("eth-mds/ricu")
```

Alternatively, the package can be installed from CRAN by using the `install.packages()` command. Using **ricu**, the user can download and set up the five large ICU datasets from North America and Europe. Data itself, however, is not part of **ricu** and while the supported datasets are publicly available, access to each of the data sources has to be requested separately. Four of the datasets, namely MIMIC-III, MIMIC-IV, eICU and HiRID are hosted on PhysioNet [22], while the fifth, AmsterdamUMCdb is currently distributed via a separate platform [23].

For the MIMIC-III and eICU datasets, small subsets of data are available as demo datasets that do not require credentialed access to PhysioNet. As the terms for distribution of these demo datasets are less restrictive, they can be made available as data packages **mimic.demo** and **eicu.demo**. Due to size constraints, however they are not available via CRAN, but can be installed from GitHub as:

```
install.packages(
  c("mimic.demo", "eicu.demo"),
  repos = "https://eth-mds.github.io/physionet-demo"
)
```

The demo datasets are especially handy for users who wish to investigate the capabilities of **ricu**. Demo datasets can be set up in a matter of minutes, and the user can explore much of the functionality offered by **ricu** using just the demo data.

The recommended way of setting up the full data, after data access is granted, is the following. Credentials can either be provided as environment variables (`RICU_PHYSIONET_USER` and `RICU_PHYSIONET_PASS` for access to PhysioNet data, as well as `RICU_AUMC_TOKEN` for AmsterdamUMCdb) and if the corresponding variables are unset, user input is again required in interactive sessions. For non-interactive sessions, functionality is exported such that data can be downloaded and set up ahead of first access (see `?setup_src_data` for the documentation).

Contingent on being granted access by the data owners, download requires a stable Internet connection, as well as 50 to 100 GB of temporary disk storage for unpacking and preparing the data for efficient access. In terms of permanent storage, 5 to 10 GB per dataset are required (see Table 1), while memory requirements are kept reasonably low by iterating over row-chunks for setup operations. Laptop class hardware (8-16 GB of memory) should suffice for setup and many analysis tasks which focus only on subsets of rows (and columns). Initial data source setup (depending on available download speeds and CPU/disk type) may take upwards of an hour per dataset. In Table 1 we provide a summary of the available datasets, giving the user some idea about the different data sources. A more detailed discussion of the data sources, and in particular how they are represented within **ricu**, is given in Supplementary Material B. For other data source inquiries, we refer the user to the original documentation of the datasets, or to the recent review paper which analyzes some of upsides and downsides of each of them [24].

Table 1. Comparison of datasets supported by **ricu**, highlighting some of the major similarities and distinguishing features. Values followed by parenthesized ranges represent medians and are accompanied by interquartile ranges.

	MIMIC-III	eICU	AmsterdamUMCdb	HiRID	MIMIC-IV
Number of tables	26	31	7	5	27
Disk storage [GB]	6.04	6.50	10.81	4.52	10.33
Largest table [rows]	330,712,483	151,604,232	977,625,612	776,921,131	329,499,788
Available concepts*	89	87	85	74	87
**Data collection**					
Time span	2001–2012	2014–2015	2003–2016	2008–2016	2008–2019
Country of origin	United States	United States	Netherlands	Switzerland	United States
**Admission counts**					
ICU	61,532	200,859	23,106	33,904	76,540
Hospital	57,841	166,355	-	-	69,300
Unique patients	46,476	-	20,109	-	53,150
**Stay lengths [day]**					
ICU stays	2.09 (1.11–4.48)	1.57 (0.82–2.97)	1.07 (0.84–3.67)	0.99 (0.81–2.16)	1.93 (1.09–3.73)
Hospital stays	6.57 (3.80–11.86)	5.05 (2.71–9.03)	-	-	6.62 (3.87–11.36)
**Vital signs [1/hour]**					
Heart rate	1.00 (1.00–1.02)	12.00 (12.00–12.00)	60.00 (60.00–60.00)	30.00 (30.00–60.00)	1.00 (1.00–1.00)
Mean arterial pressure	1.00 (1.00–1.33)	12.00 (4.00–12.00)	60.00 (60.00–60.00)	30.00 (30.00–60.00)	1.00 (1.00–1.02)
**Lab tests [1/day]**					
Bilirubin	1.00 (0.86–1.38)	1.00 (0.91–1.20)	1.00 (0.33–1.06)	1.00 (0.98–1.04)	1.00 (0.94–1.30)
Lactate	4.72 (1.84–10.75)	3.78 (1.54–6.67)	7.42 (4.30–14.12)	4.66 (2.96–7.96)	4.68 (2.29–9.47)

* These values represent the number of atomic concepts per data source. Additionally, 27 recursive concepts are available, which build on data source specific atomic concepts in a source agnostic manner (see Supplement C for details).

Adding External Datasets

As mentioned earlier, **ricu** is designed with extensibility in mind, also allowing the user to add their own data, including data that is not publicly available. However, the addition of custom datasets requires several configuration steps, which are outlined in the Adding external datasets section of Supplementary Material D.

Concepts

Concepts are the main building blocks of the **ricu** package. Most of the concepts that are ready-to-use with **ricu** are grounded in the OMOP Vocabulary, but not all, since some are not covered by the vocabulary. Concepts can be loaded using the integer OMOP concept ID, or using the abbreviated concept name strings, for fostering efficiency of code writing. Currently, there are 119 concepts available in **ricu**, which fall into four broad groups. We refer to this set of concepts as the **ricu** *dictionary*. In Table 2 we provide an overview of the groups, categories within each group, and the number of available concepts in each category. Data on any of the concepts can be loaded using the

Group	Category	Count
Physiology	blood gas	10
	chemistry	21
	hematology	20
	neurological	7
	output	2
	respiratory	10
	vitals	6
Treatment	medications	17
	microbiology	1
Demographics	demographics	6
Outcomes	outcome	19

Table 2. Number of currently available concepts in **ricu** grouped by category.

`load_concepts()` function, which is the main workhorse of the package. The function outputs three possible data types, which are the following:

(i) Data with no time stamps (such as the concept sex, OMOP concept ID 37116947), outputs the type `id_tbl`, e.g.:

```
# An ‘id_tbl‘: 3 x 2
# Id var:      ‘icustay_id‘
  icustay_id omop_37116947
       <int> <chr>
1     201006 Male
2     201204 Female
3     203766 Female
```

This output format has only the patient identifier, and the value of the concept.

(ii) Data with time stamps (such as say lactate levels, OMOP concept ID 4191725, but can also be loaded using the abbreviation `"lact"`, with the result shown below), outputs the type `ts_tbl`, formatted as:

```
# A ‘ts_tbl‘: 3 x 3
# Id var:     ‘icustay_id‘
# Units:      ‘lact‘ [mmol/L]
# Index var:  ‘charttime‘ (1 hours)
  icustay_id charttime  lact
       <int> <drtn>    <dbl>
1     201006 -58 hours   1.7
2     201006 -10 hours   1.8
3     201006   0 hours   2.2
```

This output format has the patient identifier, the time stamp, and the value of the concept.

(iii) Data with start and end time stamps (such as mechanical ventilation), outputs the type `win_tbl`:

```
# A ‘win_tbl‘:  3 x 4
# Id var:       ‘admissionid‘
# Index var:    ‘start‘ (1 hours)
# Duration var: ‘dur_var‘
  admissionid start   dur_var  mech_vent
```

```
        <int> <drtn>  <drtn>   <chr>
1           0 5 hours 810 mins invasive
2           1 0 hours 405 mins invasive
3           2 0 hours 159 mins invasive
```

This output format has the patient identifier, the time stamp of the start point, the duration of the concept, and the value of the concept.

Using the `load_concepts()` function, the user can also load multiple concepts at the same time. In that case, one of the above data types is returned. If the data is queried using OMOP concept IDs, the column names of the resulting output will contain the corresponding IDs, whereas if abbreviated string names are used, the output will have these as column names. For a more detailed explanation of the output returned by `load_concepts()` in case of loading multiple concepts, we refer the reader to Supplementary Material C.

Adding concepts

When interested in using a concept, the user can first try to investigate whether the concept is available within **ricu**. If this is not the case, **ricu** provides a mechanism for extending the dictionary with arbitrary user-specified concepts. The recommended way of specifying a concept is using a JSON-formatted text file. As an illustration, we show how the concept heart rate (which already exists in the dictionary), could be specified by the user, if it were not available. The following entry would create a concept `hr` for the MIMIC-III demo dataset:

```
{
  "hr": {
    "unit": ["bpm", "/min"],
    "min": 0,
    "max": 300,
    "omopid": 4239408,
    "description": "heart rate",
    "category": "routine vital signs",
    "sources": {
      "mimic_demo": [
        {
          "ids": [211, 220045],
          "table": "chartevents",
          "sub_var": "itemid"
        }
      ]
    }
  }
}
```

After creating such a JSON-formatted text file, the user needs to set the environment variable `RICU_CONFIG_PATH` to the folder where the file is located. Upon doing so, the user can use the `load_concepts()` function to load the concept `hr`. The heart rate values would be loaded from the `chartevents` table in MIMIC-III, by taking the subset of rows for which the value in the column `itemid` equals to either 221 or 220045, which are the codes corresponding to heart rate. Values lower than 0 and higher than 300 would be removed from the returned result (the `max` and `min` fields are not mandatory in general). An alternative method for specifying a concept would be to use the `concept()` and `item()` functionality. This alternative method is illustrated in a worked example in the Results section.

In general, especially when pre-processing is required, specifying a concept can be more involved. In this case, the user can input a *callback* function which performs arbitrary pre-processing on the data that is loaded, before it is returned by `load_concepts()`. For more details, we refer the reader to Supplementary Material C, where concept loading and specification is explained in more depth.

Other functionality

Beyond the data download and setup functionality, and the `load_concepts()` function, the **ricu** package also has a number of helper functions for working with the data. For example, there is functionality for handling time series data, taking minima or maxima over time windows, imputing values, and many more. In the interest of space, we do not go into full detail about this additional supporting functionality, but rather refer the user to the package documentation.

Results

The capabilities of the **ricu** package are perhaps best illustrated by using it for two case studies. We apply **ricu** to first study the association of lactate and mortality, and then study the association of insulin dosage and diabetes. The two examples are intended to showcase specific aspects of the package discussed in the Findings section.

Lactate and mortality

First, the association of lactate levels and mortality is investigated. This problem has been studied in the literature, and it is widely accepted that both static and dynamic lactate indices are associated with increased mortality [25, 26, 27]. In order to understand the relationship of lactate and mortality, we fitted a time-varying proportional hazards Cox model [28, 29] to the time series data which includes the SOFA score (as a general predictor of illness severity) and the lactate values. The analysis was performed on the MIMIC-III demo data. Furthermore, for the sake of this example, the patient cohort consists of patients between 20 and 90 years of age.

```
R> src <- "mimic_demo"
R>
R> cohort <- load_concepts("age", src, verbose = FALSE)
R>
R> dat <- load_concepts(c("lact", "death", "sofa"), src,
+                       patient_ids = cohort[age > 20 &
+                                            age < 90, ],
+                       verbose = FALSE)
R>
R> dat <- dat[,
+   head(.SD, n = match(TRUE, death, .N)),
+   by = c(id_vars(dat))
+ ]
R>
R> dat <- fill_gaps(dat)
R>
R> dat <- replace_na(dat, c(NA, FALSE),
+                    type = c("locf", "const"),
+                    vars = c("lact", "death"),
+                    by = id_vars(dat), by_ref = TRUE)
R>
R> cox_mod <- coxph(
+   Surv(charttime - 1L, charttime, death) ~ lact + sofa,
+   data = dat
+ )
```

After loading the data, some minor pre-processing is still required before modeling: first, data is filtered such that only data up to (and including) the hour in which the `death` flag switches to `TRUE`

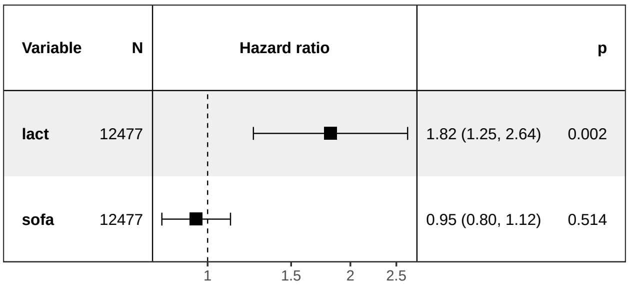

Figure 1. Forest plot for the hazard ratio of lactate levels obtained on the MIMIC-III demo dataset.

is used. After this, missing values for `lact` are imputed using a last observation carry forward (LOCF) scheme (observing the patient grouping) and missing `death` values are set to `FALSE` (contained in the usage of the `replace_na()` function). The resulting fit of the proportional hazards model can be visualized in Figure 1.

A simple exploration already shows that the increased values of lactate are associated with mortality, even after adjusting for the SOFA score. Using the abstraction provided by **ricu**, this analysis can also be applied to other datasets with minimal effort, by simply changing the `src` argument and re-running the code above.

Diabetes and insulin treatment

For the next example, now using the full MIMIC-III data, we illustrate the usage of comorbidities and treatment related information. Firstly, we focus on the amount of insulin administered to patients in the first 24 hours from their ICU admission. As the **ricu** dictionary already contains an insulin concept called `ins`, we introduce the `ins24` concept, which builds on top of the existing `ins`, and represents the total amount of insulin administered in first 24 hours of ICU stay. This can be implemented by specifying the `ins24` concept as a *recursive concept* (`rec_cncpt`), requesting data from `ins`.

In order to calculate the total amount of administered insulin, it is required to change the default aggregation method from `median()` to `sum()`. Failing to do so would yield under-reported values whenever several insulin administrations fall within a given time-step. The callback function `ins_cb()` shown below is then inserted into the loading process, performing the pre-processing steps outlined above: first data is subsetted to fall into the first 24 hours of ICU admissions, followed by binning of summed values.

```
R> ins_breaks <- c(0, 1, 10, 20, 40, Inf)
R>
R> ins_cb <- function(ins, ...) {
+
+   day_one <- function(x) x >= hours(0L) & x <= hours(24L)
+   idx_var <- index_var(ins)
+   ids_var <- id_vars(ins)
+
+   ins <- ins[
+     day_one(get(idx_var)), list(ins24 = sum(ins)),
+     by = c(ids_var)
+   ]
+   ins <- ins[,
+     ins24 := list(cut(ins24, breaks = ins_breaks,
+                       right = FALSE))
+   ]
+   ins
+ }
```

The `ins24` concept can then be specified by adding the following entry into the JSON-formatted configuration file specifying additional concepts:

```
{
  "ins24": {
    "concepts": "ins",
    "description": "Insulin in first 24 hours",
    "callback": "ins_cb",
    "class": "rec_cncpt",
    "aggregate": "sum",
    "target": "id_tbl"
  }
}
```

which then makes the concept `ins24` available for the `load_concepts()` function. Next, we want to obtain the diabetic status of the patients in the database. The diabetes concept can be implemented as `lgl_cncpt` (as diabetes is a binary variable), by matching International Classification of Diseases 9 (ICD-9) codes using a regular expression. For creating the required callback function, which produces a logical vector, the exported function factory `transform_fun()` can be employed, coupled with a function like `grep_diab()` (matching all ICD-9 codes starting with 250), performing the desired transformation.

```
R> grep_diab <- function(x) {
+   grepl("^250\\.?[0-9]{2}$", x)
+ }
```

To specify the diabetes concept `diab` into the dictionary, the following entry is added to the configuration file:

```
{
  "diab": {
    "class": "lgl_cncpt",
    "description": "diabetes status",
    "target": "id_tbl",
    "sources": {
      "mimic": [
        {
          "table": "diagnoses_icd",
          "class": "col_itm",
          "callback": "transform_fun(grep_diab)"
        }
      ]
    }
  }
}
```

Finally, with both the `ins24` and `diab` concepts ready, we can perform an analysis of the association of diabetes with insulin administration, using the following code:

```
R> src <- "mimic"
R> diab  <- item(src, table = "diagnoses_icd",
+               callback = transform_fun(grep_diab),
+               class = "col_itm")
R>
R> diab  <- concept("diab", diab, "diabetes",
+                   target = "id_tbl", class = "lgl_cncpt")
R>
R> dat <- load_concepts(c("ins24", "diab"), src,
+                       id_type = "icustay")
R> dat <- replace_na(dat, "[0,1)", vars = "ins24")
```

Following this, the difference between the two groups can be visualized with a histogram over the binned insulin administration values, as shown in Figure 2. The plot suggests that during the first day of ICU stay, perhaps unsurprisingly, diabetic patients are more likely to receive a large amount of administered insulin (p-value < 0.001 using a χ^2-test for the null hypothesis that diabetes and insulin amount are independent).

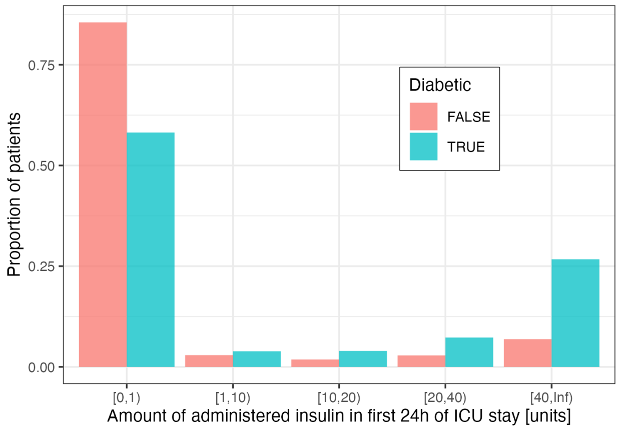

Figure 2. Association of diabetes and the amount of insulin administered in the first 24 hours of ICU stay in the MIMIC-III dataset. The difference between the diabetic and non-diabetic groups is statistically significant for each insulin bin ($p < 0.001$ for each χ^2-test).

Discussion and Conclusion

We developed the **ricu** R-package for handling five large publicly available ICU datasets. It allows in its current form the user to load various clinical concepts through a unified interface, abstracting out dataset specifics, and shielding the user from the data cleaning and data mining process. The available concepts include physiological, demographic, and treatment-related information, and also some important clinical outcomes. Most of the concepts in the package are grounded in the OMOP Vocabulary, although we found that this is a non-trivial effort in practice, due to the lack of documentation across different data sources, and the heterogeneous nature of the different datasets. Importantly, the package we developed was designed with extensibility in mind. In particular, the user can extend the existing concept dictionary of the package, and also add external data sources, which can then be analyzed in combination with the existing publicly available data.

The main implication of our work is that a software tool is now available which aims to shield researchers from developing computational infrastructure completely anew. In particular, the extensible design implies that researchers can leverage the infrastructure of the **ricu** package even if their question of interest requires clinical concepts which are beyond the current scope of the package. Furthermore, researchers using their own data now have an opportunity to import their data into the **ricu** package, and analyze the data in conjunction with the publicly available data, thereby allowing them to test the external validity of their scientific findings. Another implication of our work is that developing guidelines for publicly sharing ICU data would be a worthwhile undertaking. Currently, the publicly available datasets are very heterogeneous, both in terms of design and available documentation, and rarely provide explicit mappings to existing vocabularies/ontologies, such as the one used by OMOP.

To the best of our knowledge, there are no previous software tools that serve the same purpose as the **ricu** package. The most similar work to ours, in which the authors aim to abstract away some of the dataset specifics, has so far exclusively focused on the MIMIC dataset[19]. Therefore, we hope that the **ricu** package can become a community-wide effort which enables additions or modifications of new or existing concepts. Furthermore, **ricu** easily allows for the addition of new datasets, and we therefore expect the number of available data sources to grow over time.

The main strength of the **ricu** package is the ease with which it allows the user to set up and analyze multiple datasets through a unified interface. Furthermore, the breadth of the datasets supported by the package, which originate from three countries on two different continents, is another strength of our work. This allows users to test their hypothesis in different populations, making their findings more likely to be biologically plausible and relevant. One current limitation of the **ricu** package is that the dictionary available with the package is not comprehensive. However, we expect to develop the package further and hope that the **ricu** dictionary will grow over time. One final limitation we mention is that not every concept in the **ricu** dictionary is mapped to the OMOP Vocabulary, since some of the concepts do not exist therein. Therefore, mapping them onto the ontology is left for future work, once they become available within it.

In conclusion, the **ricu** package, developed in this manuscript, allows the user to load 119 clinical concepts from the MIMIC-III, MIMIC-IV, eICU, AUMCdb, and HiRID datasets. The package can now be used by the research community to save time by circumventing the process of developing computational infrastructure, to foster reproducible research, and to allow researchers to test the external validity and robustness of their scientific hypotheses and models.

Availability of source code

- Project name: ricu
- Project home page: https://github.com/eth-mds/ricu
- RRID: SCR_023318
- Biotools: ricu
- Operating system(s): Platform independent
- Programming language: R
- Other requirements: -
- License: GNU GPL3

Data availability

All of the data handled by the **ricu** package is publicly available. The MIMIC-III and eICU demo datasets can be accessed immediately from Github by running `remotes::install_github("eth-mds/eicu.demo")`.

For the full datasets, access requests are needed to respective dataset owners. Four of the five datasets, namely MIMIC-III, MIMIC-IV, eICU and HiRID are hosted on PhysioNet [22], while the fifth, AmsterdamUMCdb is currently distributed via a separate platform [23]. Snapshots of our code and other data further supporting this work are openly available in the GigaScience repository, GigaDB [30].

Declarations

List of abbreviations

API: Application Programming Interface; AUMC: Amsterdam University Medical Center; BIDMC: Beth Israel Deaconess Medical Center; CRAN: Comprehensive R Archive Network; DB: Database; EHR: Electronic Health Records; eICU: Electronic Intensive Care Unit; HiRID: High Time-Resolution ICU Dataset; ICD: International Classification of Diseases; ICU: Intensive Care Unit; ID: Identifier; ML: Machine Learning; OMOP: Observational Medical Outcomes Partnership; SOFA: Sequential Organ Failure Assessment.

Competing Interests

The authors declare no Competing Interests.

Funding

Nicolas Bennett, Drago Plečko, Nicolai Meinshausen and Peter Bühlmann were supported by grant #2017-110 of the Strategic Focal Area ”Personalized Health and Related Technologies (PHRT)” of the ETH Domain for the SPHN/PHRT Driver Project ”Personalized Swiss Sepsis Study”. Ida-Fong Ukor was supported by her institution.

Author's Contributions

NB and DP designed the software package. NB implemented the software package. DP and NB wrote the manuscript. IU provided supporting clinical knowledge throughout the package development. NM and PB revised the manuscript.

Acknowledgements

The authors would like to thank Rinaldo Bellomo for many inspiring discussions that initiated their work in the field of critical care medicine.

References

1. Evans RS. Electronic Health Records: Then, Now, and in the Future. Yearbook of Medical Informatics 2016;25(S 01):48–61.
2. Jiang F, Jiang Y, Zhi H, Dong Y, Li H, Ma S, et al. Artificial Intelligence in Healthcare: Past, Present and Future. Stroke and Vascular Neurology 2017;2(4):230–243.
3. Pickering BW, Gajic O, Ahmed A, Herasevich V, Keegan MT. Data Utilization for Medical Decision Making at the Time of Patient Admission to ICU. Critical Care Medicine 2013;41(6):1502–1510.
4. Desautels T, Calvert J, Hoffman J, Jay M, Kerem Y, Shieh L, et al. Prediction of Sepsis in the Intensive Care Unit with Minimal Electronic Health Record Data: A Machine Learning Approach. JMIR Medical Informatics 2016;4(3):e28.
5. Nemati S, Holder A, Razmi F, Stanley MD, Clifford GD, Buchman TG. An Interpretable Machine Learning Model for Accurate Prediction of Sepsis in the ICU. Critical Care Medicine 2018;46(4):547–553.
6. Futoma J, Hariharan S, Sendak M, Brajer N, Clement M, Bedoya A, et al., An Improved Multi-Output Gaussian Process RNN With Real-Time Validation for Early Sepsis Detection; 2017. ArXiv:1708.05894.
7. Kam HJ, Kim HY. Learning Representations for the Early Detection of Sepsis With Deep Neural Networks. Computers in Biology and Medicine 2017;89:248–255.
8. Fleuren LM, Klausch TLT, Zwager CL, Schoonmade LJ, Guo T, Roggeveen LF, et al. Machine Learning for the Prediction of Sepsis: A Systematic Review and Meta-Analysis of Diagnostic Test Accuracy. Intensive Care Medicine 2020;46(3):383–400.
9. Johnson AE, Pollard TJ, Shen L, Li-wei HL, Feng M, Ghassemi M, et al. MIMIC-III, A Freely Accessible Critical Care Database. Scientific Data 2016;3:160035.
10. Wong A, Otles E, Donnelly JP, Krumm A, McCullough J, DeTroyer-Cooley O, et al. External Validation of a Widely Implemented Proprietary Sepsis Prediction Model in Hospitalized Patients. JAMA Internal Medicine 2021;.
11. Singer M, Deutschman CS, Seymour CW, Shankar-Hari M, Annane D, Bauer M, et al. The Third International Consensus Definitions for Sepsis and Septic Shock (Sepsis-3). JAMA 2016 feb;315(8):801–810.
12. Wang RZ, Sun CH, Schroeder PH, Ameko MK, Moore CC, Barnes LE. Predictive Models of Sepsis in Adult ICU Patients. In: 2018 IEEE International Conference on Healthcare Informatics (ICHI) Institute of Electrical and Electronics Engineers; 2018. p. 390–391.
13. Johnson AEW, Aboab J, Raffa JD, Pollard TJ, Deliberato RO, Celi LA, et al. A Comparative Analysis of Sepsis Identification Methods in an Electronic Database. Critical Care Medicine 2018 Apr;46(4):494–499.
14. Pollard TJ, Johnson AE, Raffa JD, Celi LA, Mark RG, Badawi O. The eICU Collaborative Research Database, A Freely Available Multi-Center Database for Critical Care Research. Scientific Data 2018;5:180178.
15. Faltys M, Zimmermann M, Lyu X, Hüser M, Hyland SL, Rätsch G, et al., HiRID, A High Time-Resolution ICU Dataset (Version 1.1.1); 2021. PhysioNet.
16. Thoral PJ, Peppink JM, Driessen RH, Sijbrands EJG, Kompanje EJO, Kaplan L, et al. Sharing ICU Patient Data Responsibly Under the Society of Critical Care Medicine/European Society of Intensive Care Medicine Joint Data Science Collaboration: The Amsterdam University Medical Centers Database (AmsterdamUMCdb) Example. Critical Care Medicine 2021;Latest Articles.
17. Johnson A, Bulgarelli L, Pollard T, Horng S, Celi LA, Mark R, MIMIC-IV (Version 1.0); 2021. PhysioNet.
18. Adibuzzaman M, Musselman K, Johnson A, Brown P, Pitluk Z, Grama A. Closing the Data Loop: An Integrated Open Access Analysis Platform for the MIMIC Database. In: 2016 Computing in Cardiology Conference (CinC) Institute of Electrical and Electronics Engineers; 2016. p. 137–140.
19. Wang S, McDermott MB, Chauhan G, Ghassemi M, Hughes MC, Naumann T. MIMIC-Extract: A Data Extraction, Preprocessing, and Representation Pipeline for MIMIC-III. In: Proceedings of the ACM Conference on Health, Inference, and Learning Association for Computing Machinery; 2020. p. 222–235.
20. Glicksberg BS, Oskotsky B, Giangreco N, Thangaraj PM, Rudrapatna V, Datta D, et al. ROMOP: a light-weight R package for interfacing with OMOP-formatted electronic health record data. JAMIA open 2019;2(1):10–14.
21. Glicksberg BS, Oskotsky B, Thangaraj PM, Giangreco N, Badgeley MA, Johnson KW, et al. PatientExploreR: an extensible application for dynamic visualization of patient clinical history from electronic health records in the OMOP common data model. Bioinformatics 2019;35(21):4515–4518.
22. Goldberger AL, Amaral LAN, Glass L, Hausdorff JM, Ivanov PC, Mark RG, et al. PhysioBank, PhysioToolkit and PhysioNet. Circulation 2000;101(23):e215–e220.
23. Amsterdam University Medical Center's Database Collaborators and the SCCM/ESICM Joint Data Science Task Force, Amsterdam University Medical Center Database; 2020. [Online; accessed 18-May-2023]. https://amsterdammedicaldatascience.nl/amsterdamumcdb/.
24. Sauer CM, Dam TA, Celi LA, Faltys M, de la Hoz MA, Adhikari L, et al. Systematic Review and Comparison of Publicly Available ICU Data Sets—A Decision Guide for Clinicians and Data Scientists. Critical care medicine 2022;50(6):e581–e588.
25. Haas SA, Lange T, Saugel B, Petzoldt M, Fuhrmann V, Metschke M, et al. Severe Hyperlactatemia, Lactate Clearance and Mortality in Unselected Critically Ill Patients. Intensive Care Medicine 2016;42(2):202–210.
26. Nichol A, Bailey M, Egi M, Pettila V, French C, Stachowski E, et al. Dynamic Lactate Indices as Predictors of Outcome in Critically Ill Patients. Critical Care 2011;15(5):R242.
27. Van Beest PA, Brander L, Jansen SP, Rommes JH, Kuiper MA, Spronk PE. Cumulative Lactate and Hospital Mortality in ICU Patients. Annals of Intensive Care 2013;3(1):6.
28. Therneau TM, Grambsch PM. Modeling Survival Data:

Extending the Cox Model. New York: Springer-Verlag; 2000.
29. Therneau TM. A Package for Survival Analysis in R; 2021, https://CRAN.R-project.org/package=survival, R package version 3.2-11.
30. Nicolas B, Drago P, Ida-Fong U, Meinshausen Nicolai, Peter B, Supporting data for "ricu: R's Interface to Intensive Care Data". GigaScience Database; 2023. http://gigadb.org/dataset/102392.

Manuscript

Click here to access/download;Manuscript;ricu_GigaScienceR3.pdf

GigaScience, 2023, 1–8

doi: xx.xxxx/xxxx
Manuscript in Preparation
Technical Note

TECHNICAL NOTE

ricu: R's Interface to Intensive Care Data

Nicolas Bennett[1,†], Drago Plečko[1,†,*], Ida-Fong Ukor[2], Nicolai Meinshausen[1] and Peter Bühlmann[1]

[1]Seminar for Statistics, ETH Zürich and [2]Department of Anaesthesiology and Perioperative Medicine, Monash Health

*Correspondence email address: drago.plecko@stat.math.ethz.ch
†Contributed equally.

Abstract

Objective: To develop a unified framework for analyzing data from five large, publicly available ICU datasets.
Findings: Using three American (MIMIC-III, MIMIC-IV, eICU), and two European (AUMCdb, HiRID) databases, we constructed a mapping for each database to a set of clinically relevant concepts, which are grounded in the OMOP Vocabulary wherever possible. Furthermore, we performed synchronization in the units of measurement, and data type representation. On top of this, we built functionality which allows the user to download, set up, and load data from all of the five databases, through a unified Application Programming Interface (API). The resulting **ricu** R-package represents the computational infrastructure for handling publicly available ICU datasets and its latest release allows the user to load 119 existing clinical concepts from the five data sources.
Conclusion: The **ricu** R-package (available on Github and CRAN) is the first tool that enables users to analyze publicly available ICU datasets simultaneously (datasets are available upon request from respective owners). Such an interface saves researchers time when analyzing ICU data, and helps reproducibility. We hope that **ricu** can become a community-wide effort, so that data harmonization is not repeated by each research group separately. One current limitation is that concepts were added on a case-to-case basis, and therefore the resulting dictionary of concepts is not comprehensive. Further work is needed to make the dictionary comprehensive.

Keywords: Intensive Care Medicine; Electronic Health Records; Computational Methods

Introduction

Collection of electronic health records has seen a significant rise in recent years [1], opening up opportunities and providing the grounds for a large body of data-driven research oriented towards helping clinicians in decision-making and therefore improving patient care and health outcomes [2]. While growing amounts of collected patient data might not be easily utilized by intensivists for decision-making [3], this poses an opportunity for the application of machine learning (ML) methods.

One example of a problem that has received much attention from the ML community is early prediction of sepsis in the intensive care unit (ICU) [4, 5, 6, 7]. Interestingly, there is evidence that a large proportion of the publications on this topic are based on the same dataset [8], the Medical Information Mart for Intensive Care III (MIMIC-III) [9], showing a systematic lack of external validation. This issue has recently again been highlighted by a study demonstrating poor performance in external validation of a widely adopted sepsis prediction model [10].

Contributing to this problem might well be the lack of computational infrastructure for handling multiple datasets. The MIMIC-III dataset consists of 26 different tables containing about 20GB of data. While much work and care has gone into data pre-processing in order to provide a self-contained ready-to-use data resource with MIMIC-III, seemingly simple tasks such as computing a Sepsis-3 label [11] remain non-trivial efforts[1]. This is only exacerbated when aiming to co-integrate multiple different datasets of this form, spanning hospitals and even countries, in order to capture effects of differing practice and demographics.

Given the somewhat specific focus of ICU data, it may come

1 There is considerable heterogeneity in the number of patients satisfying the Sepsis-3 criterion [11] among studies investigating MIMIC-III. Reported Sepsis-3 prevalence ranges from 11.3% [4], over 23.9% [5] and 25.4% [12], up to 49.1% [13]. While some of this variation may be explained by differing patient inclusion criteria, differences in label implementation must also contribute significantly.

Compiled on: May 18, 2023.
Draft manuscript prepared by the author.

Key Points

- The **ricu** R-package is the first tool that enables users to analyze five large ICU datasets through a unified interface,
- **ricu** currently supports 119 clinical concepts across MIMIC-III, MIMIC-IV, eICU, HiRID, and AUMCdb datasets,
- **ricu** allows for easy addition of user-specified concepts and datasets, integrating them with the existing infrastructure.

as a surprise as to how heterogeneous the currently available datasets are. In MIMIC-III and HiRID, for example, time-stamps are reported as absolute times (albeit randomly shifted due to data privacy concerns), whereas eICU and AmsterdamUMCdb use relative times (with origins being admission times). Another example involves different types of patient identifiers and their use among datasets. Common to all is the notion of an ICU admission identifier (ID), but apart from that, the amount of available information varies: While ICU (and hospital) readmissions for a given patient can be identified in some, this is not possible in other datasets. Furthermore, use of identifier systems might not be consistent over tables. In MIMIC-III, for example, some tables refer to ICU stay IDs while others use hospital stay IDs, which slightly complicates data retrieval for a fixed ID system. Additionally, table layouts vary (*long* versus *wide* data arrangement) and data organization in general is far from consistent over datasets.

In light of the above described background, the aim of **ricu** is to provide computational infrastructure allowing users to investigate complex research questions in the context of critical care medicine as easily as possible, by introducing a unified interface to a heterogeneous set of data sources. The package enables users to write dataset-agnostic code which can simplify implementation and shorten the time necessary for prototyping code querying different datasets. In its current form, the package handles five large-scale, publicly available intensive care databases out of the box: MIMIC-III [9] from the Beth Israel Deaconess Medical Center (BIDMC) in Boston, Massachusetts, the eICU Collaborative Research Database [14], containing data collected from 208 hospitals across the United States, the High Time Resolution ICU Dataset (HiRID) [15] from the Department of Intensive Care Medicine of the Bern University Hospital, Switzerland, the Amsterdam University Medical Center Database (AmsterdamUMCdb) [16] from the Amsterdam University Medical Center, and MIMIC-IV [17], again using data from BIDMC. Furthermore, **ricu** was designed with extensibility in mind such that adding new public and/or private user-provided datasets is possible. Being implemented in R, a programming language popular among statisticians and data analysts, it is our hope to contribute to accessible and reproducible research by using a familiar environment and requiring only few system dependencies, thereby considerably simplifying setup.

To our knowledge, infrastructure that provides a common interface to multiple ICU datasets is a novel contribution. While there have been efforts [18, 19] attempting to abstract away some specifics of a dataset, these have so far exclusively focused on MIMIC-III, the most popular of public ICU datasets, and have not been designed with dataset interoperability in mind. It is also worth mentioning software packages **ROMOP** [20] and **PatientExploreR** [21] that offer some useful infrastructure for data compatible with the Observational Medical Outcomes Partnership (OMOP) common data model, but the heterogeneity of the datasets supported by **ricu** limits the ability to organize them according to such a data model.

Findings

The **ricu** package can be accessed from either Github or CRAN, and the lastest development version can be installed by running:

```
devtools::install_github("eth-mds/ricu")
```

Alternatively, the package can be installed from CRAN by using the `install.packages()` command. Using **ricu**, the user can download and set up the five large ICU datasets from North America and Europe. Data itself, however, is not part of **ricu** and while the supported datasets are publicly available, access to each of the data sources has to be requested separately. Four of the datasets, namely MIMIC-III, MIMIC-IV, eICU and HiRID are hosted on PhysioNet [22], while the fifth, AmsterdamUMCdb is currently distributed via a separate platform [23].

For the MIMIC-III and eICU datasets, small subsets of data are available as demo datasets that do not require credentialed access to PhysioNet. As the terms for distribution of these demo datasets are less restrictive, they can be made available as data packages **mimic.demo** and **eicu.demo**. Due to size constraints, however they are not available via CRAN, but can be installed from GitHub as:

```
install.packages(
  c("mimic.demo", "eicu.demo"),
  repos = "https://eth-mds.github.io/physionet-demo"
)
```

The demo datasets are especially handy for users who wish to investigate the capabilities of **ricu**. Demo datasets can be set up in a matter of minutes, and the user can explore much of the functionality offered by **ricu** using just the demo data.

The recommended way of setting up the full data, after data access is granted, is the following. Credentials can either be provided as environment variables (`RICU_PHYSIONET_USER` and `RICU_PHYSIONET_PASS` for access to PhysioNet data, as well as `RICU_AUMC_TOKEN` for AmsterdamUMCdb) and if the corresponding variables are unset, user input is again required in interactive sessions. For non-interactive sessions, functionality is exported such that data can be downloaded and set up ahead of first access (see `?setup_src_data` for the documentation).

Contingent on being granted access by the data owners, download requires a stable Internet connection, as well as 50 to 100 GB of temporary disk storage for unpacking and preparing the data for efficient access. In terms of permanent storage, 5 to 10 GB per dataset are required (see Table 1), while memory requirements are kept reasonably low by iterating over row-chunks for setup operations. Laptop class hardware (8-16 GB of memory) should suffice for setup and many analysis tasks which focus only on subsets of rows (and columns). Initial data source setup (depending on available download speeds and CPU/disk type) may take upwards of an hour per dataset. In Table 1 we provide a summary of the available datasets, giving the user some idea about the different data sources. A more detailed discussion of the data sources, and in particular how they are represented within **ricu**, is given in Supplementary Material B. For other data source inquiries, we refer the user to the original documentation of the datasets, or to the recent review paper which analyzes some of upsides and downsides of each of them [24].

Table 1. Comparison of datasets supported by **ricu**, highlighting some of the major similarities and distinguishing features. Values followed by parenthesized ranges represent medians and are accompanied by interquartile ranges.

	MIMIC-III	eICU	AmsterdamUMCdb	HiRID	MIMIC-IV
Number of tables	26	31	7	5	27
Disk storage [GB]	6.04	6.50	10.81	4.52	10.33
Largest table [rows]	330,712,483	151,604,232	977,625,612	776,921,131	329,499,788
Available concepts*	89	87	85	74	87
**Data collection**					
Time span	2001–2012	2014–2015	2003–2016	2008–2016	2008–2019
Country of origin	United States	United States	Netherlands	Switzerland	United States
**Admission counts**					
ICU	61,532	200,859	23,106	33,904	76,540
Hospital	57,841	166,355	-	-	69,300
Unique patients	46,476	-	20,109	-	53,150
**Stay lengths [day]**					
ICU stays	2.09 (1.11–4.48)	1.57 (0.82–2.97)	1.07 (0.84–3.67)	0.99 (0.81–2.16)	1.93 (1.09–3.73)
Hospital stays	6.57 (3.80–11.86)	5.05 (2.71–9.03)	-	-	6.62 (3.87–11.36)
**Vital signs [1/hour]**					
Heart rate	1.00 (1.00–1.02)	12.00 (12.00–12.00)	60.00 (60.00–60.00)	30.00 (30.00–60.00)	1.00 (1.00–1.00)
Mean arterial pressure	1.00 (1.00–1.33)	12.00 (4.00–12.00)	60.00 (60.00–60.00)	30.00 (30.00–60.00)	1.00 (1.00–1.02)
**Lab tests [1/day]**					
Bilirubin	1.00 (0.86–1.38)	1.00 (0.91–1.20)	1.00 (0.33–1.06)	1.00 (0.98–1.04)	1.00 (0.94–1.30)
Lactate	4.72 (1.84–10.75)	3.78 (1.54–6.67)	7.42 (4.30–14.12)	4.66 (2.96–7.96)	4.68 (2.29–9.47)

* These values represent the number of atomic concepts per data source. Additionally, 27 recursive concepts are available, which build on data source specific atomic concepts in a source agnostic manner (see Supplement C for details).

Adding External Datasets

As mentioned earlier, **ricu** is designed with extensibility in mind, also allowing the user to add their own data, including data that is not publicly available. However, the addition of custom datasets requires several configuration steps, which are outlined in the Adding external datasets section of Supplementary Material D.

Concepts

Concepts are the main building blocks of the **ricu** package. Most of the concepts that are ready-to-use with **ricu** are grounded in the OMOP Vocabulary, but not all, since some are not covered by the vocabulary. Concepts can be loaded using the integer OMOP concept ID, or using the abbreviated concept name strings, for fostering efficiency of code writing. Currently, there are 119 concepts available in **ricu**, which fall into four broad groups. We refer to this set of concepts as the **ricu** *dictionary*. In Table 2 we provide an overview of the groups, categories within each group, and the number of available concepts in each category. Data on any of the concepts can be loaded using the `load_concepts()` function, which is the main workhorse of the package. The function outputs three possible data types, which are the following:

Group	Category	Count
Physiology	blood gas	10
	chemistry	21
	hematology	20
	neurological	7
	output	2
	respiratory	10
	vitals	6
Treatment	medications	17
	microbiology	1
Demographics	demographics	6
Outcomes	outcome	19

Table 2. Number of currently available concepts in **ricu** grouped by category.

(i) Data with no time stamps (such as the concept sex, OMOP concept ID 37116947), outputs the type `id_tbl`, e.g.:

```
# An ‘id_tbl‘: 3 x 2
# Id var:      ‘icustay_id‘
  icustay_id omop_37116947
       <int> <chr>
1     201006 Male
2     201204 Female
3     203766 Female
```

This output format has only the patient identifier, and the value of the concept.

(ii) Data with time stamps (such as say lactate levels, OMOP concept ID 4191725, but can also be loaded using the abbreviation `"lact"`, with the result shown below), outputs the type `ts_tbl`, formatted as:

```
# A ‘ts_tbl‘: 3 x 3
# Id var:     ‘icustay_id‘
# Units:      ‘lact‘ [mmol/L]
# Index var:  ‘charttime‘ (1 hours)
  icustay_id charttime  lact
       <int> <drtn>    <dbl>
1     201006 -58 hours   1.7
2     201006 -10 hours   1.8
3     201006   0 hours   2.2
```

This output format has the patient identifier, the time stamp, and the value of the concept.

(iii) Data with start and end time stamps (such as mechanical ventilation), outputs the type `win_tbl`:

```
# A ‘win_tbl‘:  3 x 4
# Id var:       ‘admissionid‘
# Index var:    ‘start‘ (1 hours)
# Duration var: ‘dur_var‘
  admissionid start   dur_var  mech_vent
```

```
        <int> <drtn>  <drtn>   <chr>
1           0 5 hours 810 mins invasive
2           1 0 hours 405 mins invasive
3           2 0 hours 159 mins invasive
```

This output format has the patient identifier, the time stamp of the start point, the duration of the concept, and the value of the concept.

Using the `load_concepts()` function, the user can also load multiple concepts at the same time. In that case, one of the above data types is returned. If the data is queried using OMOP concept IDs, the column names of the resulting output will contain the corresponding IDs, whereas if abbreviated string names are used, the output will have these as column names. For a more detailed explanation of the output returned by `load_concepts()` in case of loading multiple concepts, we refer the reader to Supplementary Material C.

Adding concepts

When interested in using a concept, the user can first try to investigate whether the concept is available within **ricu**. If this is not the case, **ricu** provides a mechanism for extending the dictionary with arbitrary user-specified concepts. The recommended way of specifying a concept is using a JSON-formatted text file. As an illustration, we show how the concept heart rate (which already exists in the dictionary), could be specified by the user, if it were not available. The following entry would create a concept `hr` for the MIMIC-III demo dataset:

```
{
  "hr": {
    "unit": ["bpm", "/min"],
    "min": 0,
    "max": 300,
    "omopid": 4239408,
    "description": "heart rate",
    "category": "routine vital signs",
    "sources": {
      "mimic_demo": [
        {
          "ids": [211, 220045],
          "table": "chartevents",
          "sub_var": "itemid"
        }
      ]
    }
  }
}
```

After creating such a JSON-formatted text file, the user needs to set the environment variable `RICU_CONFIG_PATH` to the folder where the file is located. Upon doing so, the user can use the `load_concepts()` function to load the concept `hr`. The heart rate values would be loaded from the `chartevents` table in MIMIC-III, by taking the subset of rows for which the value in the column `itemid` equals to either 221 or 220045, which are the codes corresponding to heart rate. Values lower than 0 and higher than 300 would be removed from the returned result (the `max` and `min` fields are not mandatory in general). An alternative method for specifying a concept would be to use the `concept()` and `item()` functionality. This alternative method is illustrated in a worked example in the Results section.

In general, especially when pre-processing is required, specifying a concept can be more involved. In this case, the user can input a *callback* function which performs arbitrary pre-processing on the data that is loaded, before it is returned by `load_concepts()`. For more details, we refer the reader to Supplementary Material C, where concept loading and specification is explained in more depth.

Other functionality

Beyond the data download and setup functionality, and the `load_concepts()` function, the **ricu** package also has a number of helper functions for working with the data. For example, there is functionality for handling time series data, taking minima or maxima over time windows, imputing values, and many more. In the interest of space, we do not go into full detail about this additional supporting functionality, but rather refer the user to the package documentation.

Results

The capabilities of the **ricu** package are perhaps best illustrated by using it for two case studies. We apply **ricu** to first study the association of lactate and mortality, and then study the association of insulin dosage and diabetes. The two examples are intended to showcase specific aspects of the package discussed in the Findings section.

Lactate and mortality

First, the association of lactate levels and mortality is investigated. This problem has been studied in the literature, and it is widely accepted that both static and dynamic lactate indices are associated with increased mortality [25, 26, 27]. In order to understand the relationship of lactate and mortality, we fitted a time-varying proportional hazards Cox model [28, 29] to the time series data which includes the SOFA score (as a general predictor of illness severity) and the lactate values. The analysis was performed on the MIMIC-III demo data. Furthermore, for the sake of this example, the patient cohort consists of patients between 20 and 90 years of age.

```
R> src <- "mimic_demo"
R>
R> cohort <- load_concepts("age", src, verbose = FALSE)
R>
R> dat <- load_concepts(c("lact", "death", "sofa"), src,
+                       patient_ids = cohort[age > 20 &
+                                              age < 90, ],
+                       verbose = FALSE)
R>
R> dat <- dat[,
+   head(.SD, n = match(TRUE, death, .N)),
+   by = c(id_vars(dat))
+ ]
R>
R> dat <- fill_gaps(dat)
R>
R> dat <- replace_na(dat, c(NA, FALSE),
+                    type = c("locf", "const"),
+                    vars = c("lact", "death"),
+                    by = id_vars(dat), by_ref = TRUE)
R>
R> cox_mod <- coxph(
+   Surv(charttime - 1L, charttime, death) ~ lact + sofa,
+   data = dat
+ )
```

After loading the data, some minor pre-processing is still required before modeling: first, data is filtered such that only data up to (and including) the hour in which the `death` flag switches to `TRUE`

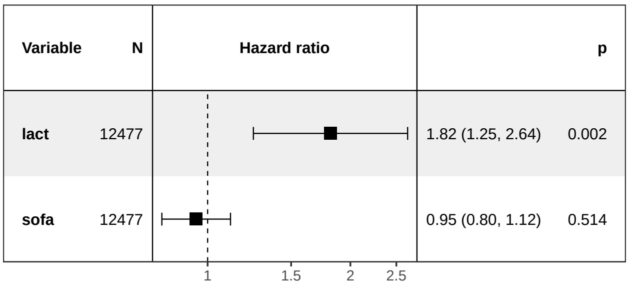

Figure 1. Forest plot for the hazard ratio of lactate levels obtained on the MIMIC-III demo dataset.

is used. After this, missing values for `lact` are imputed using a last observation carry forward (LOCF) scheme (observing the patient grouping) and missing `death` values are set to `FALSE` (contained in the usage of the `replace_na()` function). The resulting fit of the proportional hazards model can be visualized in Figure 1.

A simple exploration already shows that the increased values of lactate are associated with mortality, even after adjusting for the SOFA score. Using the abstraction provided by **ricu**, this analysis can also be applied to other datasets with minimal effort, by simply changing the `src` argument and re-running the code above.

Diabetes and insulin treatment

For the next example, now using the full MIMIC-III data, we illustrate the usage of comorbidities and treatment related information. Firstly, we focus on the amount of insulin administered to patients in the first 24 hours from their ICU admission. As the **ricu** dictionary already contains an insulin concept called `ins`, we introduce the `ins24` concept, which builds on top of the existing `ins`, and represents the total amount of insulin administered in first 24 hours of ICU stay. This can be implemented by specifying the `ins24` concept as a *recursive concept* (`rec_cncpt`), requesting data from `ins`.

In order to calculate the total amount of administered insulin, it is required to change the default aggregation method from `median()` to `sum()`. Failing to do so would yield under-reported values whenever several insulin administrations fall within a given time-step. The callback function `ins_cb()` shown below is then inserted into the loading process, performing the preprocessing steps outlined above: first data is subsetted to fall into the first 24 hours of ICU admissions, followed by binning of summed values.

```
R> ins_breaks <- c(0, 1, 10, 20, 40, Inf)
R>
R> ins_cb <- function(ins, ...) {
+
+   day_one <- function(x) x >= hours(0L) & x <= hours(24L)
+   idx_var <- index_var(ins)
+   ids_var <- id_vars(ins)
+
+   ins <- ins[
+     day_one(get(idx_var)), list(ins24 = sum(ins)),
+     by = c(ids_var)
+   ]
+   ins <- ins[,
+     ins24 := list(cut(ins24, breaks = ins_breaks,
+                       right = FALSE))
+   ]
+   ins
+ }
```

The `ins24` concept can then be specified by adding the following entry into the JSON-formatted configuration file specifying additional concepts:

```
{
  "ins24": {
    "concepts": "ins",
    "description": "Insulin in first 24 hours",
    "callback": "ins_cb",
    "class": "rec_cncpt",
    "aggregate": "sum",
    "target": "id_tbl"
  }
}
```

which then makes the concept `ins24` available for the `load_concepts()` function. Next, we want to obtain the diabetic status of the patients in the database. The diabetes concept can be implemented as `lgl_cncpt` (as diabetes is a binary variable), by matching International Classification of Diseases 9 (ICD-9) codes using a regular expression. For creating the required callback function, which produces a logical vector, the exported function factory `transform_fun()` can be employed, coupled with a function like `grep_diab()` (matching all ICD-9 codes starting with 250), performing the desired transformation.

```
R> grep_diab <- function(x) {
+   grepl("^250\\.?[0-9]{2}$", x)
+ }
```

To specify the diabetes concept `diab` into the dictionary, the following entry is added to the configuration file:

```
{
  "diab": {
    "class": "lgl_cncpt",
    "description": "diabetes status",
    "target": "id_tbl",
    "sources": {
      "mimic": [
        {
          "table": "diagnoses_icd",
          "class": "col_itm",
          "callback": "transform_fun(grep_diab)"
        }
      ]
    }
  }
}
```

Finally, with both the `ins24` and `diab` concepts ready, we can perform an analysis of the association of diabetes with insulin administration, using the following code:

```
R> src <- "mimic"
R> diab  <- item(src, table = "diagnoses_icd",
+                callback = transform_fun(grep_diab),
+                class = "col_itm")
R>
R> diab  <- concept("diab", diab, "diabetes",
+                    target = "id_tbl", class = "lgl_cncpt")
R>
R> dat <- load_concepts(c("ins24", "diab"), src,
+                        id_type = "icustay")
R> dat <- replace_na(dat, "[0,1)", vars = "ins24")
```

Following this, the difference between the two groups can be visualized with a histogram over the binned insulin administration values, as shown in Figure 2. The plot suggests that during the first day of ICU stay, perhaps unsurprisingly, diabetic patients are more likely to receive a large amount of administered insulin (p-value < 0.001 using a χ^2-test for the null hypothesis that diabetes and insulin amount are independent).

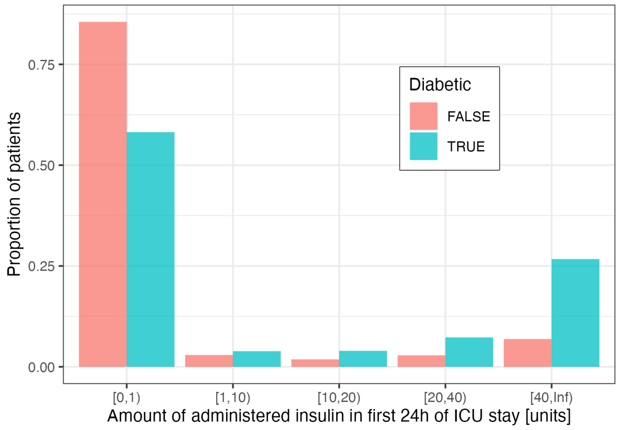

Figure 2. Association of diabetes and the amount of insulin administered in the first 24 hours of ICU stay in the MIMIC-III dataset. The difference between the diabetic and non-diabetic groups is statistically significant for each insulin bin ($p < 0.001$ for each χ^2-test).

Discussion and Conclusion

We developed the **ricu** R-package for handling five large publicly available ICU datasets. It allows in its current form the user to load various clinical concepts through a unified interface, abstracting out dataset specifics, and shielding the user from the data cleaning and data mining process. The available concepts include physiological, demographic, and treatment-related information, and also some important clinical outcomes. Most of the concepts in the package are grounded in the OMOP Vocabulary, although we found that this is a non-trivial effort in practice, due to the lack of documentation across different data sources, and the heterogeneous nature of the different datasets. Importantly, the package we developed was designed with extensibility in mind. In particular, the user can extend the existing concept dictionary of the package, and also add external data sources, which can then be analyzed in combination with the existing publicly available data.

The main implication of our work is that a software tool is now available which aims to shield researchers from developing computational infrastructure completely anew. In particular, the extensible design implies that researchers can leverage the infrastructure of the **ricu** package even if their question of interest requires clinical concepts which are beyond the current scope of the package. Furthermore, researchers using their own data now have an opportunity to import their data into the **ricu** package, and analyze the data in conjunction with the publicly available data, thereby allowing them to test the external validity of their scientific findings. Another implication of our work is that developing guidelines for publicly sharing ICU data would be a worthwhile undertaking. Currently, the publicly available datasets are very heterogeneous, both in terms of design and available documentation, and rarely provide explicit mappings to existing vocabularies/ontologies, such as the one used by OMOP.

To the best of our knowledge, there are no previous software tools that serve the same purpose as the **ricu** package. The most similar work to ours, in which the authors aim to abstract away some of the dataset specifics, has so far exclusively focused on the MIMIC dataset[19]. Therefore, we hope that the **ricu** package can become a community-wide effort which enables additions or modifications of new or existing concepts. Furthermore, **ricu** easily allows for the addition of new datasets, and we therefore expect the number of available data sources to grow over time.

The main strength of the **ricu** package is the ease with which it allows the user to set up and analyze multiple datasets through a unified interface. Furthermore, the breadth of the datasets supported by the package, which originate from three countries on two different continents, is another strength of our work. This allows users to test their hypothesis in different populations, making their findings more likely to be biologically plausible and relevant. One current limitation of the **ricu** package is that the dictionary available with the package is not comprehensive. However, we expect to develop the package further and hope that the **ricu** dictionary will grow over time. One final limitation we mention is that not every concept in the **ricu** dictionary is mapped to the OMOP Vocabulary, since some of the concepts do not exist therein. Therefore, mapping them onto the ontology is left for future work, once they become available within it.

In conclusion, the **ricu** package, developed in this manuscript, allows the user to load 119 clinical concepts from the MIMIC-III, MIMIC-IV, eICU, AUMCdb, and HiRID datasets. The package can now be used by the research community to save time by circumventing the process of developing computational infrastructure, to foster reproducible research, and to allow researchers to test the external validity and robustness of their scientific hypotheses and models.

Availability of source code

- Project name: ricu
- Project home page: https://github.com/eth-mds/ricu
- RRID: SCR_023318
- Biotools: ricu
- Operating system(s): Platform independent
- Programming language: R
- Other requirements: -
- License: GNU GPL3

Data availability

All of the data handled by the **ricu** package is publicly available. The MIMIC-III and eICU demo datasets can be accessed immediately from Github by running `remotes::install_github("eth-mds/eicu.demo")`.

For the full datasets, access requests are needed to respective dataset owners. Four of the five datasets, namely MIMIC-III, MIMIC-IV, eICU and HiRID are hosted on PhysioNet [22], while the fifth, AmsterdamUMCdb is currently distributed via a separate platform [23]. Snapshots of our code and other data further supporting this work are openly available in the GigaScience repository, GigaDB [30].

Declarations

List of abbreviations

API: Application Programming Interface; AUMC: Amsterdam University Medical Center; BIDMC: Beth Israel Deaconess Medical Center; CRAN: Comprehensive R Archive Network; DB: Database; EHR: Electronic Health Records; eICU: Electronic Intensive Care Unit; HiRID: High Time-Resolution ICU Dataset; ICD: International Classification of Diseases; ICU: Intensive Care Unit; ID: Identifier; ML: Machine Learning; OMOP: Observational Medical Outcomes Partnership; SOFA: Sequential Organ Failure Assessment.

Competing Interests

The authors declare no Competing Interests.

Funding

Nicolas Bennett, Drago Plečko, Nicolai Meinshausen and Peter Bühlmann were supported by grant #2017-110 of the Strategic Focal Area "Personalized Health and Related Technologies (PHRT)" of the ETH Domain for the SPHN/PHRT Driver Project "Personalized Swiss Sepsis Study". Ida-Fong Ukor was supported by her institution.

Author's Contributions

NB and DP designed the software package. NB implemented the software package. DP and NB wrote the manuscript. IU provided supporting clinical knowledge throughout the package development. NM and PB revised the manuscript.

Acknowledgements

The authors would like to thank Rinaldo Bellomo for many inspiring discussions that initiated their work in the field of critical care medicine.

References

1. Evans RS. Electronic Health Records: Then, Now, and in the Future. Yearbook of Medical Informatics 2016;25(S 01):48–61.
2. Jiang F, Jiang Y, Zhi H, Dong Y, Li H, Ma S, et al. Artificial Intelligence in Healthcare: Past, Present and Future. Stroke and Vascular Neurology 2017;2(4):230–243.
3. Pickering BW, Gajic O, Ahmed A, Herasevich V, Keegan MT. Data Utilization for Medical Decision Making at the Time of Patient Admission to ICU. Critical Care Medicine 2013;41(6):1502–1510.
4. Desautels T, Calvert J, Hoffman J, Jay M, Kerem Y, Shieh L, et al. Prediction of Sepsis in the Intensive Care Unit with Minimal Electronic Health Record Data: A Machine Learning Approach. JMIR Medical Informatics 2016;4(3):e28.
5. Nemati S, Holder A, Razmi F, Stanley MD, Clifford GD, Buchman TG. An Interpretable Machine Learning Model for Accurate Prediction of Sepsis in the ICU. Critical Care Medicine 2018;46(4):547–553.
6. Futoma J, Hariharan S, Sendak M, Brajer N, Clement M, Bedoya A, et al., An Improved Multi-Output Gaussian Process RNN With Real-Time Validation for Early Sepsis Detection; 2017. ArXiv:1708.05894.
7. Kam HJ, Kim HY. Learning Representations for the Early Detection of Sepsis With Deep Neural Networks. Computers in Biology and Medicine 2017;89:248–255.
8. Fleuren LM, Klausch TLT, Zwager CL, Schoonmade LJ, Guo T, Roggeveen LF, et al. Machine Learning for the Prediction of Sepsis: A Systematic Review and Meta-Analysis of Diagnostic Test Accuracy. Intensive Care Medicine 2020;46(3):383–400.
9. Johnson AE, Pollard TJ, Shen L, Li-wei HL, Feng M, Ghassemi M, et al. MIMIC-III, A Freely Accessible Critical Care Database. Scientific Data 2016;3:160035.
10. Wong A, Otles E, Donnelly JP, Krumm A, McCullough J, DeTroyer-Cooley O, et al. External Validation of a Widely Implemented Proprietary Sepsis Prediction Model in Hospitalized Patients. JAMA Internal Medicine 2021;.
11. Singer M, Deutschman CS, Seymour CW, Shankar-Hari M, Annane D, Bauer M, et al. The Third International Consensus Definitions for Sepsis and Septic Shock (Sepsis-3). JAMA 2016 feb;315(8):801–810.
12. Wang RZ, Sun CH, Schroeder PH, Ameko MK, Moore CC, Barnes LE. Predictive Models of Sepsis in Adult ICU Patients. In: 2018 IEEE International Conference on Healthcare Informatics (ICHI) Institute of Electrical and Electronics Engineers; 2018. p. 390–391.
13. Johnson AEW, Aboab J, Raffa JD, Pollard TJ, Deliberato RO, Celi LA, et al. A Comparative Analysis of Sepsis Identification Methods in an Electronic Database. Critical Care Medicine 2018 Apr;46(4):494–499.
14. Pollard TJ, Johnson AE, Raffa JD, Celi LA, Mark RG, Badawi O. The eICU Collaborative Research Database, A Freely Available Multi-Center Database for Critical Care Research. Scientific Data 2018;5:180178.
15. Faltys M, Zimmermann M, Lyu X, Hüser M, Hyland SL, Rätsch G, et al., HiRID, A High Time-Resolution ICU Dataset (Version 1.1.1); 2021. PhysioNet.
16. Thoral PJ, Peppink JM, Driessen RH, Sijbrands EJG, Kompanje EJO, Kaplan L, et al. Sharing ICU Patient Data Responsibly Under the Society of Critical Care Medicine/European Society of Intensive Care Medicine Joint Data Science Collaboration: The Amsterdam University Medical Centers Database (AmsterdamUMCdb) Example. Critical Care Medicine 2021;Latest Articles.
17. Johnson A, Bulgarelli L, Pollard T, Horng S, Celi LA, Mark R, MIMIC-IV (Version 1.0); 2021. PhysioNet.
18. Adibuzzaman M, Musselman K, Johnson A, Brown P, Pitluk Z, Grama A. Closing the Data Loop: An Integrated Open Access Analysis Platform for the MIMIC Database. In: 2016 Computing in Cardiology Conference (CinC) Institute of Electrical and Electronics Engineers; 2016. p. 137–140.
19. Wang S, McDermott MB, Chauhan G, Ghassemi M, Hughes MC, Naumann T. MIMIC-Extract: A Data Extraction, Preprocessing, and Representation Pipeline for MIMIC-III. In: Proceedings of the ACM Conference on Health, Inference, and Learning Association for Computing Machinery; 2020. p. 222–235.
20. Glicksberg BS, Oskotsky B, Giangreco N, Thangaraj PM, Rudrapatna V, Datta D, et al. ROMOP: a light-weight R package for interfacing with OMOP-formatted electronic health record data. JAMIA open 2019;2(1):10–14.
21. Glicksberg BS, Oskotsky B, Thangaraj PM, Giangreco N, Badgeley MA, Johnson KW, et al. PatientExploreR: an extensible application for dynamic visualization of patient clinical history from electronic health records in the OMOP common data model. Bioinformatics 2019;35(21):4515–4518.
22. Goldberger AL, Amaral LAN, Glass L, Hausdorff JM, Ivanov PC, Mark RG, et al. PhysioBank, PhysioToolkit and PhysioNet. Circulation 2000;101(23):e215–e220.
23. Amsterdam University Medical Center's Database Collaborators and the SCCM/ESICM Joint Data Science Task Force, Amsterdam University Medical Center Database; 2020. [Online; accessed 18-May-2023]. https://amsterdammedicaldatascience.nl/amsterdamumcdb/.
24. Sauer CM, Dam TA, Celi LA, Faltys M, de la Hoz MA, Adhikari L, et al. Systematic Review and Comparison of Publicly Available ICU Data Sets—A Decision Guide for Clinicians and Data Scientists. Critical care medicine 2022;50(6):e581–e588.
25. Haas SA, Lange T, Saugel B, Petzoldt M, Fuhrmann V, Metschke M, et al. Severe Hyperlactatemia, Lactate Clearance and Mortality in Unselected Critically Ill Patients. Intensive Care Medicine 2016;42(2):202–210.
26. Nichol A, Bailey M, Egi M, Pettila V, French C, Stachowski E, et al. Dynamic Lactate Indices as Predictors of Outcome in Critically Ill Patients. Critical Care 2011;15(5):R242.
27. Van Beest PA, Brander L, Jansen SP, Rommes JH, Kuiper MA, Spronk PE. Cumulative Lactate and Hospital Mortality in ICU Patients. Annals of Intensive Care 2013;3(1):6.
28. Therneau TM, Grambsch PM. Modeling Survival Data:

Extending the Cox Model. New York: Springer-Verlag; 2000.

29. Therneau TM. A Package for Survival Analysis in R; 2021, https://CRAN.R-project.org/package=survival, R package version 3.2-11.
30. Nicolas B, Drago P, Ida-Fong U, Meinshausen Nicolai, Peter B, Supporting data for ”ricu: R's Interface to Intensive Care Data”. GigaScience Database; 2023. http://gigadb.org/dataset/102392.

Click here to access/download
Supplementary Material
supplementA.pdf

Click here to access/download
Supplementary Material
supplementB.pdf

Click here to access/download
Supplementary Material
supplementC.pdf

Click here to access/download
Supplementary Material
supplementD.pdf
