# Supplementary material for: ricu: R’s interface to intensive care data
Source: Gigascience. 2023 Jun 15;12:giad041. doi: 10.1093/gigascience/giad041 (PMC10268223; doi:10.1093/gigascience/giad041)
Supplement: giad041_Supplemental_Files [file giad041_supplemental_files.zip › supplementA.pdf]

Supplement A: Quick Start

The following list gives a quick outline of the steps required for setting up and starting to use **ricu**, alongside some section references on where to find further details. A more comprehensive version of this overview is available as a separate vignette within the package.

1. Package installation:

   - Installation from CRAN as `install.packages("ricu")` provides the most recently released version of **ricu**.
   - Alternatively, the latest development version is available from GitHub by running `remotes::install_github("eth-mds/ricu")` (to do so, you may first need to install the `remotes` package from CRAN using `install.packages("remotes")`).

2. Requesting access to datasets and data source setup:

   - Demo datasets can be set up by installing the data packages `mimic.demo` and/or `eicu.demo` from GitHub using `install.packages()`, as shown in the Findings section.
   - The complete MIMIC-III, eICU, HiRID and MIMIC-IV datasets can be accessed by registering and setting up a credentialed account at PhysioNet.
   - Access to AmsterdamUMCdb can be requested via the Amsterdam Medical Data Science Website.
   - The obtained credentials can be configured for PhysioNet datasets by setting environment variables `RICU_PHYSIONET_USER` and `RICU_PHYSIONET_PASS`, while the download token for AmsterdamUMCdb can be set as `RICU_AUMC_TOKEN`.
   - Datasets are downloaded and set up either automatically upon the first access attempt or manually by running `setup_data_src()`; the environment variable `RICU_DATA_PATH` can be set to control data location.
   - Dataset availability can be queried by calling `src_data_avail()`.

   A more detailed description of the supported datasets is given in Findings, summarized in Table 1, while Supplementary Material B provides implementation details, elaborating on how datasets are represented in code.

3. Loading of data corresponding to clinical concepts using `load_concepts()`:

   - Currently, over 100 data concepts are available for the five supported datasets (see the functions `concept_availability()`/`explain_dictionary()` for names, availability etc.).
   - For example, glucose and age data can be loaded by passing the argument `c("age", "glu")` to `load_concepts()` (or by passing `c(4314456L, 4144235L)` when loading using the OMOP concept IDs).

   Supplementary Material C goes into more detail on how data concepts are represented within **ricu**, and an overview of the pre-configured concepts is available from the Ready-to-use concepts section therein.

4. Extending the concept dictionary:

   - Data concepts can be specified in code using the constructors `concept()`/`item()` or `new_concept()`/`new_item()`.
   - For session persistence, data concepts can also be specified as JSON formatted objects.

- JSON-based concept dictionaries can either extend or replace others and they can be pointed to by setting the environment variable `RICU_CONFIG_PATH`.

The JSON format used to encode data concepts is discussed in more detail in Concept specification section of Supplement C.

5. Adding new datasets:

   - A JSON-based dataset configuration file is required, from which the configuration objects described in the Data source configuration section are created.
   - In order for concepts to be available from the new dataset, the dictionary requires extension by adding new data items.

   Further information about adding a new dataset is available in the Adding external datasets section of Supplement D. Some code used when AmsterdamUMCdb was not yet fully integrated with **ricu** is available from GitHub and is used for demonstration purposes to set up AmsterdamUMCdb as an external dataset `aumc_ext`.
