# Supplementary material for: ricu: R’s interface to intensive care data
Source: Gigascience. 2023 Jun 15;12:giad041. doi: 10.1093/gigascience/giad041 (PMC10268223; doi:10.1093/gigascience/giad041)
Supplement: giad041_Supplemental_Files [file giad041_supplemental_files.zip › supplementB.pdf]

Supplement B: Data Sources

MIMIC-III

The Medical Information Mart for Intensive Care III (MIMIC-III) represents the third iteration of the arguably most influential initiative for collecting and providing large-scale ICU data to the public. The dataset comprises de-identified health related data of roughly 46,000 patients admitted to critical care units of Beth Israel Deaconess Medical Center (BIDMC) during the years 2001-2012. Amounting to just over 61,000 individual ICU admission, data is available on demographics, routine vital sign measurements (at approximately 1 hour resolution), laboratory tests, medication, as well as critical care procedures, organized as a 26-table relational structure.

```
R> mimic
```

```
<mimic_env[26]>
        admissions             callout          caregivers         chartevents
      [58,976 x 19]       [34,499 x 24]         [7,567 x 4] [330,712,483 x 15]
         cptevents               d_cpt     d_icd_diagnoses    d_icd_procedures
     [573,146 x 12]          [134 x 9]        [14,567 x 4]          [3,882 x 4]
           d_items          d_labitems      datetimeevents       diagnoses_icd
      [12,487 x 10]           [753 x 6]    [4,485,937 x 14]       [651,047 x 5]
          drgcodes            icustays     inputevents_cv      inputevents_mv
      [125,557 x 8]       [61,532 x 12]  [17,527,935 x 22]    [3,618,991 x 31]
         labevents microbiologyevents         noteevents        outputevents
   [27,854,055 x 9]      [631,726 x 16]    [2,083,180 x 11]    [4,349,218 x 13]
          patients       prescriptions procedureevents_mv      procedures_icd
        [46,520 x 8]    [4,156,450 x 19]     [258,066 x 25]       [240,095 x 5]
          services           transfers
        [73,343 x 6]      [261,897 x 13]
```

One thing of note from a data-organizational perspective is that a change in electronic health care systems occurred in 2008. Owing to this, roughly 38,000 ICU admissions spanning the years 2001 though 2008 are documented using the CareVue system, while for 2008 and onwards, data was extracted from the MetaVision clinical information system. Item identifiers differ between the two systems, requiring queries to consider both ID mappings (heart rate for example being available both as `itemid` number `211` for CareVue and `220045` for MetaVision) as does documentation of infusions and other procedures that are considered as input events (cf., `inputevents_cv` and `inputevents_mv` tables). Especially with respect to such input event data, MetaVision generally provides data of superior quality.

In terms of patient identifiers, MIMIC-III allows for identifying both individual patients (`subject_id`) across hospital admissions (`hadm_id`) and for connecting ICU (re)admissions (`icustay_id`) to hospital admissions. Using the respective one-to-many relationships, **ricu** can retrieve patient data using any of the above IDs, irrespective of how the raw data is organized.

eICU

Unlike the single-center focus of other datasets, the eICU Collaborative Research Database constitutes an amalgamation of data from critical care units of over 200 hospitals throughout the continental United States. Large-scale data collected via the Philips eICU program, which provides telehealth infrastructure for intensive

care units, is available from the Philips eICU Research Institute (eRI), albeit neither publicly nor freely. Only data corresponding to roughly 200,000 ICU admissions, sampled from a larger population of over 3 million ICU admissions and stratified by hospital, is being made available via PhysioNet. Patients with discharge dates in 2014 or 2015 were considered, with stays in low acuity units being removed.

```
R> eicu
```

```
<eicu_env[31]>
          admissiondrug                 admissiondx                   allergy
          [874,920 x 14]                [626,858 x 6]             [251,949 x 13]
           apacheapsvar          apachepatientresult               apachepredvar
          [171,177 x 26]               [297,064 x 23]             [171,177 x 51]
   careplancareprovider                 careplaneol             careplangeneral
           [502,765 x 8]                  [1,433 x 5]             [3,115,018 x 6]
           careplangoal careplaninfectiousdisease                   customlab
           [504,139 x 7]                  [8,056 x 8]                [1,082 x 7]
              diagnosis                    hospital                infusiondrug
         [2,710,672 x 7]                    [208 x 4]             [4,803,719 x 9]
           intakeoutput                         lab                  medication
       [12,030,289 x 12]          [39,132,531 x 10]           [7,301,853 x 15]
               microlab                        note            nurseassessment
            [16,996 x 7]              [2,254,179 x 8]           [15,602,498 x 8]
              nursecare                nursecharting                pasthistory
         [8,311,132 x 8]            [151,604,232 x 8]           [1,149,180 x 8]
                patient                physicalexam             respiratorycare
          [200,859 x 29]              [9,212,316 x 6]             [865,381 x 34]
  respiratorycharting                     treatment             vitalaperiodic
        [20,168,176 x 7]              [3,688,745 x 5]          [25,075,074 x 13]
          vitalperiodic
     [146,671,642 x 19]
```

The data is organized into 31 tables and includes patient demographics, routine vital signs, laboratory measurements, medication administrations, admission diagnoses, as well as treatment information. Owing to the wide range of hospitals participating in this data collection initiative, spanning small, rural, non-teaching health centers with fewer than 100 beds to large teaching hospitals with an excess of 500 beds, data availability varies. Even if data was being recorded at the bedside it might end up missing from the eICU dataset due to technical limitations of the collection process. As for patient identifiers, while it is possible to link ICU admissions corresponding to the same hospital stay, it is not possible to identify patients across hospital stays.

Data resolution again varies considerably over included variables. The `vitalperiodic` table stands out as one of the few examples of a *wide* table organization (laying out variables as columns), as opposed to the *long* presentation (following an entity–attribute–value model) of most other tables containing patient measurement data. The average time step in `vitalperiodic` is around 5 minutes, but data missingness ranges from around 1% for heart rate and pulse oximetry to roughly 10% for respiration rate and up to 80% for systemic and 90% for pulmonary artery blood pressure measurements, therefore giving approximately hourly resolution for such variables.

HiRID

Developed for early prediction of circulatory failure[27], the High Time Resolution ICU Dataset (HiRID) contains data on almost 34,000 admissions to the Department of Intensive Care Medicine of the Bern University Hospital, Switzerland, an interdisciplinary 60-bed unit. Given the clear focus on a concrete

application during data collection, this dataset is the most limited in terms of data breadth, which is also reflected in a comparatively simple data layout comprising only 5 tables[†].

```
R> hirid
```

```
<hirid_env[5]>
          general      observations           ordinal            pharma
     [33,905 x 5] [776,921,131 x 8]          [72 x 3] [16,270,399 x 14]
        variables
        [712 x 5]
```

Collected during the period of January 2008 through June 2016, roughly 700 distinct variables covering routine vital signs, diagnostic test results and treatment parameters are available with variables monitored at the bedside being recorded with two minute time resolution. In terms of demographic information and patient identifier systems however, the data is limited. It is not possible to identify subsequent ICU admissions corresponding to the same patient and apart from patient age, sex, weight and height, very little information is available to characterize patients. There is no medical history, no admission diagnoses, only in-ICU mortality information, no unstructured patient data and no information on patient discharge. Furthermore, data on body fluid sampling has been omitted, complicating for example the construction of a Sepsis-3 label[11].

AmsterdamUMCdb

As a second European dataset, also focusing on increased time-resolution over the US datasets, AmsterdamUMCdb has been made available in late 2019, containing data on over 23,000 intensive care unit and high dependency unit admissions of adult patients during the years 2003 through 2016. The department of Intensive Care at Amsterdam University Medical Center is a mixed medical-surgical ICU with 32 bed intensive care and 12 bed high dependency units with an average of 1000-2000 yearly admissions. Covering middle ground between the US datasets and HiRID in terms of breadth of included data, while providing a maximal time-resolution of 1 minute, AmsterdamUMCdb constitutes a well organized high quality ICU data resource organized succinctly as a 7-table relational structure.

```
R> aumc
```

```
<aumc_env[7]>
         admissions            drugitems       freetextitems         listitems
      [23,106 x 19]     [4,907,269 x 31]      [651,248 x 11]  [30,744,065 x 11]
       numericitems procedureorderitems        processitems
 [977,625,612 x 15]      [2,188,626 x 8]        [256,715 x 6]
```

For data anonymization purposes, demographic information such as patient weight, height and age only available as binned variables instead of raw numeric values. Apart from this, there is information on patient origin, mortality, admission diagnoses, as well as numerical measurements including vital parameters, lab results, outputs from drains and catheters, information on administered medication, and other medical procedures. In terms of patient identifiers, it is possible to link ICU admissions corresponding to the same individual, but it is not possible to identify separate hospital admissions.

[†]The data is available in three states: as raw data and in two intermediary preprocessing stages explained in[27]. While **ricu** focuses exclusively on raw data, the *merged* stage represents a selection of variables that were deemed most predictive for determining circulatory failure, which are then merged into 18 meta-variables, representing different clinical concepts. Time stamps in *merged* data are left unchanged, yielding irregular time series, whereas for the *imputed* stage, data is down-sampled to a 5 minute grid and missing values are imputed using a scheme discussed in[27].

MIMIC-IV

The most recently released dataset and next iteration in the MIMIC line of datasets, MIMIC-IV, has recently been released as first stable version[17] and support in **ricu** is available as dataset `miiv`. Compared to MIMIC-III, this release shifts focus to newer data, dropping all CareVue-documented patients and with that, patients who were admitted before 2008, while adding patients admitted up to and including 2019. The resulting dataset contains data on over 256,000 patients of which, 53,000 were admitted to ICUs, resulting in 76,000 unique ICU and almost 70,000 related hospital admissions.

```
R> miiv
```

```
<miiv_env[27]>
        admissions        chartevents            d_hcpcs    d_icd_diagnoses
    [523,740 x 15] [329,499,788 x 10]        [89,200 x 4]      [109,775 x 3]
  d_icd_procedures            d_items         d_labitems     datetimeevents
      [85,257 x 3]         [3,861 x 9]        [1,630 x 5]     [7,495,712 x 9]
     diagnoses_icd           drgcodes               emar        emar_detail
   [5,280,351 x 5]       [769,622 x 7]  [27,464,367 x 11]  [55,947,921 x 33]
       hcpcsevents           icustays        inputevents          labevents
     [160,727 x 6]        [76,540 x 8]   [9,460,658 x 26] [122,103,667 x 15]
microbiologyevents       outputevents           patients           pharmacy
  [3,397,914 x 24]     [4,457,381 x 8]       [382,278 x 6]  [14,736,386 x 27]
               poe         poe_detail      prescriptions    procedureevents
 [42,483,962 x 11]     [3,256,358 x 5]  [17,008,053 x 17]     [731,247 x 26]
    procedures_icd           services          transfers
     [779,625 x 6]       [562,892 x 5]    [2,189,535 x 7]
```

In addition to including newer ICU data, this MIMIC release puts both more emphasis on data collected outside the ICU, newly making emergency department (ED) data available. In a similar vein, the set of considered data types is also expanded by including chest X-ray (CXR) imagery directly with MIMIC data, using the same patient identifiers, while expanding the amount of unstructured text data (still to be made publicly available). Despite these promising developments, the focus of **ricu** remains on data that lies in the intersection of the supported datasets and therefore both ED and CXR data cannot be accessed by the current `miiv` implementation. Finally, documentation of medication administration has been much improved by not only reporting prescriptions, but, using an electronic Medicine Administration Record (eMAR) system, including time-stamped data on administration of individual formulary units.
