# Supplementary material for: ricu: R’s interface to intensive care data
Source: Gigascience. 2023 Jun 15;12:giad041. doi: 10.1093/gigascience/giad041 (PMC10268223; doi:10.1093/gigascience/giad041)
Supplement: giad041_Supplemental_Files [file giad041_supplemental_files.zip › supplementC.pdf]

Supplement C: Concepts

Ready-to-use concepts

The current selection of clinical concepts that is included with **ricu** covers many physiological variables that are available throughout the included datasets. Treatment-related information on the other hand, being more heterogeneous in nature and therefore harder to harmonize across datasets, has been added on an as-needed basis and therefore is more limited in breadth.

Available concepts can be enumerated using `load_dictionary()` and the utility function `explain_dictionary()` can be used to display some concept metadata.

```
R> dict <- load_dictionary(demo)
R> head(dict)
```

```
<concept[6]>
                                  abx                              adh_rate
           antibiotics <lgl_cncpt[4]>       vasopressin rate <num_cncpt[3]>
                                  adm                                   age
patient admission type <fct_cncpt[2]>            patient age <num_cncpt[2]>
                                  alb                                   alp
               albumin <num_cncpt[2]>   alkaline phosphatase <num_cncpt[2]>
```

```
R> explain_dictionary(head(dict))
```

```
      name     category            description
1      abx  medications            antibiotics
2 adh_rate  medications       vasopressin rate
3      adm demographics patient admission type
4      age demographics            patient age
5      alb    chemistry                albumin
6      alp    chemistry   alkaline phosphatase
```

The following subsections serve to introduce some of the included concepts as well as highlight limitations that come with current implementations. Grouping the available concepts by category yields the following counts

```
R> table(vapply(dict, `[[`, character(1L), "category"))
```

```
   blood gas    chemistry demographics   hematology  medications microbiology
          10           21            6           20           17            1
neurological      outcome       output  respiratory       vitals
           7           19            2           10            6
```

Physiological data

The largest and most well established group of concepts (covering more than half of all currently included concepts) includes physiological patient measurements such as routine vital signs, respiratory variables, fluid discharge amounts, as well as many kinds of laboratory tests including blood gas measurements, chemical analysis of body fluids and hematology assays.

```
R> load_concepts(c("alb", "glu"), src, interval = mins(15L),
+                verbose = FALSE)

# A `ts_tbl`: 1,965 x 4
# Id var:     `icustay_id`
# Units:      `alb` [g/dL], `glu` [mg/dL]
# Index var:  `charttime` (15 mins)
      icustay_id charttime    alb   glu
           <int> <drtn>     <dbl> <dbl>
    1     201006 -3495 mins  NA     116
    2     201006 -2745 mins  NA      83
    3     201006 -1275 mins  NA      91
    4     201006    15 mins   2.4   175
    5     201006   675 mins  NA     129
  ...
1,961     298685 15600 mins  NA     159
1,962     298685 16365 mins   2.2   153
1,963     298685 17400 mins  NA     182
1,964     298685 17595 mins  NA     122
1,965     298685 17955 mins   2.5   121
# ... with 1,955 more rows
```

Most concepts of this kind are represented by `num_cncpt` objects (see Concept specification section) with an associated unit of measurement and a range of permissible values. Data is mainly returned as `ts_tbl` objects, representing time-dependent observations. Apart from conversion to a common unit (using functionality offered by the **units** package[28] or possibly using the `convert_unit()` callback function), little has to be done in terms of preprocessing: values are simply reported at time-points rounded to the requested interval.

Patient demographics

Moving on from dynamic, time-varying patient data, this group of concepts focuses on static patient information. While the assumption of remaining constant throughout a stay is likely to hold for variables including patient sex or height this is only approximately true for others such as weight. Nevertheless, such effects are ignored and concepts of this group will be mainly returned as `id_tbl` objects with no corresponding time-stamps included.

Whenever requesting concepts which are returned with associated time-stamps (e.g., glucose) alongside time-constant data (e.g., age), merging will duplicate static data over all time-points.

```
R> load_concepts(c("age", "glu"), src, verbose = FALSE)

# A `ts_tbl`: 1,914 x 4
# Id var:     `icustay_id`
# Units:      `age` [years], `glu` [mg/dL]
# Index var:  `charttime` (1 hours)
      icustay_id charttime   age   glu
```

```
          <int> <drtn>    <dbl> <dbl>
    1     201006 -58 hours  68.9   116
    2     201006 -45 hours  68.9    83
    3     201006 -21 hours  68.9    91
    4     201006   0 hours  68.9   175
    5     201006  11 hours  68.9   129
  ...
1,910     298685 260 hours  80.1   159
1,911     298685 272 hours  80.1   153
1,912     298685 290 hours  80.1   182
1,913     298685 293 hours  80.1   122
1,914     298685 299 hours  80.1   121
# ... with 1,904 more rows
```

Despite a best-effort approach, data availability can be a limiting factor. While for physiological variables, there is good agreement even across countries, data-privacy considerations, as well as lack of a common standard for data encoding, may cause issues that are hard to resolve. In some cases, this can be somewhat mitigated while in others, this is a limitation to be kept in mind. In AmsterdamUMCdb, for example, patient age, height and weight are not available as continuous variables, but as factor variables with patients binned into groups. Such variables are then approximated by returning the respective mid-points of groups for `aumc` data[‡]. Other concepts, such as `adm` (categorizing admission types) or a potential `icd` concept (diagnoses as ICD-9 codes) can only return data if available from the data source in question. Unfortunately, neither `aumc` nor `hirid` contain ICD-9 encoded diagnoses, and in the case of `hirid`, no diagnosis information is available at all.

Treatment-related information

The largest group of concepts dealing with treatment-related information is described by the `medications` category. In addition to drug administrations, only basic ventilation information is currently provided as ready-to-use concept. Current drug-related concepts cover vasopressor administrations, as well as corticosteroids, antibiotics and dextrose infusions. While this group of concepts lends itself to use of `win_tbl` objects, a call to `load_concepts()`, requesting multiple concepts which do not all return data as `win_tbl` (while leaving the `merge` argument at default value `TRUE`), all `win_tbl` objects are converted to `ts_tbl` in order to be merged with the non-`win_tbl` objects.

Ventilation is represented by several concepts: a ventilation indicator variable (`vent_ind`), represented by a `win_tbl` object is constructed from start and end events (concepts `vent_start` and `vent_end`). This includes any kind of mechanical ventilation (invasive via an endotracheal or tracheostomy tube), as well as non-invasive ventilation via face or nasal masks. In line with other concepts belonging to this group, the current state is far from being comprehensive and expansion to further ventilation parameters is desirable.

The singular concept addressing antibiotics (`abx`) returns an indicator signaling whenever an antibiotic was administered. This includes any route of administration (intravenous, oral, topical, etc.) and does neither report dosage, nor active ingredient. Finally, vasopressor administration is reported by several concepts representing different vasoactive drugs (including dopamine, dobutamine, epinephrine, norepinephrine and vasopressin), as well as different administration aspects such as rate, duration and rate administered for at least 60 minutes, which is used in Sepsis-Related Organ Failure Assessment (SOFA) scoring[29].

```
R> load_concepts(c("abx", "vent_ind", "norepi_rate", "norepi_dur"), src,
+                verbose = FALSE)
```

[‡]Prioritizing consistency over accuracy, one could apply the same binning to datasets which report numeric values, but the concepts included with **ricu** attempt to strike a balance between consistency and amount of applied preprocessing. With the extensible architecture of data concepts, however, such categorical variants of patient demographic concepts could easily be added.

```
# A `ts_tbl`: 12,434 x 6
# Id var:     `icustay_id`
# Units:      `norepi_rate` [mcg/kg/min]
# Index var:  `startdate` (1 hours)
       icustay_id startdate abx   vent_ind norepi_rate norepi_dur
            <int> <drtn>    <lgl> <lgl>          <dbl> <drtn>
     1     201006   7 hours TRUE  NA            NA      NA hours
     2     201006   8 hours NA    TRUE          NA      60 hours
     3     201006   9 hours NA    TRUE            0.0460 NA hours
     4     201006  10 hours NA    TRUE            0.0690 NA hours
     5     201006  11 hours NA    TRUE            0.0690 NA hours
   ...
12,430     298685 612 hours NA    TRUE          NA      NA hours
12,431     298685 613 hours NA    TRUE          NA      NA hours
12,432     298685 614 hours NA    TRUE          NA      NA hours
12,433     298685 615 hours NA    TRUE          NA      NA hours
12,434     298685 616 hours NA    TRUE          NA      NA hours
# ... with 12,424 more rows
```

As cautioned in Patient demographics section, variability in data reporting across datasets can lead to issues: the `prescriptions` table included with MIMIC-III, for example, reports time-stamps as dates only, yielding a discrepancy of up to 24 hours when merged with data where time-accuracy is on the order of minutes. Another problem exists with concepts that attempt to report administration windows, as some datasets do not describe infusions with clear cut start/endpoints but rather report infusion parameters at (somewhat) regular time intervals. This can cause artifacts when the requested time step-size deviates from the dataset inherent time grid and introduces uncertainty when attempting to determine start/endpoints for creating a `win_tbl` object.

```
R> load_concepts("dex", "mimic_demo", verbose = FALSE)
```

```
# A `win_tbl`:  10 x 4
# Id var:       `icustay_id`
# Units:        `dex` [ml/hr]
# Index var:    `starttime` (1 hours)
# Duration var: `dur_var`
   icustay_id starttime dur_var       dex
        <int> <drtn>    <drtn>      <dbl>
 1     216185 129 hours   1 mins 15000
 2     249805 335 hours 660 mins     9.09
 3     253931 159 hours   1 mins   500.
 4     277238   2 hours 140 mins   100.
 5     277238   3 hours   1 mins 15000
 6     277238   5 hours   1 mins 15000
 7     285750   4 hours   1 mins 15000
 8     286072 101 hours   1 mins  7500
 9     286072 118 hours   1 mins  7500
10     286072 126 hours   1 mins  7500
```

Furthermore for a concept like dextrose administration as implemented in `dex`, where infusions are returned alongside bolus administrations, this can yield large rate values, as the returned unit is ml/hr and in this particular case, values are harmonized such that they correspond to 10% dextrose solutions. A bolus administration of 50 ml dextrose 50% will therefore be reported as 15000 ml/hr administered within 1 minute.

Outcomes

A group of more loosely associated concepts can be used to describe patient state. This includes common clinical endpoints, such as death or length of ICU stay, as well as scoring systems such as SOFA, the systemic inflammatory response syndrome SIRS;[30] criterion, the National Early Warning Score NEWS;[31] and the Modified Early Warning Score MEWS;[32].

While the more straightforward outcomes can be retrieved directly from data, clinical scores often incorporate multiple variables, based upon which a numeric score is constructed. This can typically be achieved by using concepts of type `rec_cncpt` (see Concept specification section), specifying the needed components and supplying a callback function that applies rules for score construction.

```
R> load_concepts(c("sirs", "death"), src, verbose = FALSE,
+                keep_components = TRUE)

# A `ts_tbl`: 14,295 x 8
# Id var:     `icustay_id`
# Index var:  `charttime` (1 hours)
       icustay_id charttime  sirs death temp_comp hr_comp resp_comp wbc_comp
            <int> <drtn>    <dbl> <lgl>     <int>   <int>     <int>    <int>
     1     201006 -58 hours     1 NA           NA      NA        NA        1
     2     201006 -45 hours     1 NA           NA      NA        NA        1
     3     201006 -21 hours     1 NA           NA      NA        NA        1
     4     201006 -10 hours     2 NA           NA      NA         1        1
     5     201006   0 hours     3 NA            0       1         1        1
   ...
14,291     298685 314 hours     2 NA            0       0         1        1
14,292     298685 315 hours     2 NA            0       0         1        1
14,293     298685 316 hours     2 NA            0       0         1        1
14,294     298685 317 hours     1 NA            0       0         0        1
14,295     298685 318 hours     1 TRUE          0       0        NA        1
# ... with 14,285 more rows
```

Callback functions can become rather involved (especially for more complex concepts such as SOFA) and may offer arbitrary arguments to tune their behavior. As callback functions to `rec_cncpt` objects are typically called internally from `load_concepts()`, arguments not used by `load_concepts()`, such as `keep_components` in the above example (causing not only the score column, but also individual score components to be retained) are forwarded. Therefore, some care has to be taken as when requesting multiple concepts within the same call to `load_concepts()`, while passing arguments intended for concept-level callback functions, as all involved callback functions will be called with the same forwarded arguments. When for example requesting multiple scores (such as SOFA or SIRS), it is currently not possible to enable `keep_components` for only a subset thereof. This setup consequently also requires that all involved callback functions are allowed to be called with the given set of extra arguments.

Concept specification

Just like data source configuration (as discussed in Data source configuration section), concept specification relies on JSON-formatted text files, parsed by **jsonlite**[33]. A default dictionary of concepts is included with **ricu**, containing a selection of commonly used clinical concepts. Several types of concepts exist within **ricu** and with extensibility in mind, new types can easily be added. A quick remark on terminology before diving into more details on how to specify data concepts: A *concept* corresponds to a clinical variable such as a bilirubin measurement or the ventilation status of a patient, and an *item* encodes how to retrieve data corresponding to a given concept from a data source. A *concept* therefore contains several *items* (zero, one or multiple are possible per data source).

All concepts consist of minimal metadata including a name, target class (defaults to `ts_tbl`; see Data classes section), an aggregation specification[§] and class information (`num_concept` if not otherwise specified), as well as optional `description` and `category` information. Adding to that, depending on concept class, further fields can be supplied. In the case of the most widespread concept type (`num_cncpt`; used to represent numeric data) this is `unit` which encodes one (or several synonymous) unit(s) of measurement, as well as a minimal and maximal plausible values (specified as `min` and `max`). The concept for heart rate data (`hr`) for example can be specified as (as already shown in Findings section)

```
{
  "hr": {
    "unit": ["bpm", "/min"],
    "min": 0,
    "max": 300,
    "omopid": 4239408,
    "description": "heart rate",
    "category": "routine vital signs",
    "sources": {
      "mimic_demo": [
        {
          "ids": [211, 220045],
          "table": "chartevents",
          "sub_var": "itemid"
        }
      ]
    }
  }
}
```

Metadata is used during concept loading for data-preprocessing. For numeric concepts, the specified measurement unit is compared to that of the data (if available), with messages being displayed in case of mismatches, while the range of plausible values is used to filter out measurements that fall outside the specified interval. Other types of concepts include categorical concepts (`fct_cncpt`), concepts representing binary data (`lgl_cncpt`), as well as recursive concepts (`rec_cncpt`), which build on other *atomic* concepts[¶].

Finally, the most recently added concept class, `unt_cncpt`, inheriting from `num_cncpt`, aims to simplify manual conversion to target units, leveraging capabilities provided by the **units** package. For this to work, both source and target units have to be recognized and convertible (as reported by `units::ud_are_convertible()`). Measurement units that are not available by default can be registered using `units::install_unit()`.

Specification of how data can be retrieved from a data source is encoded by data *items*. Lists of data items (associated with data source names) are provided as `sources` element. For the demo datasets corresponding to eICU and MIMIC-III, heart rate data retrieval is specified as

```
{
  "eicu_demo": [
    {
      "table": "vitalperiodic",
```

[§]Every concept needs a default aggregation method which can be used during data loading to return data that is unique per key (either per `id_vars` group or per combination of `id_vars` and `index_var`) otherwise down-stream merging of multiple concepts is ill-defined. The aggregation default can be manually overridden during loading or automatically, by specification as part of a `rec_cncpt` object. If no aggregation method is explicitly indicated the global default is `first()` for character, `median()` for numeric and `any()` for logical vectors.

[¶]An example for a recursive concept is the PaO_2/FiO_2 ratio, used for instance to assess patients with acute respiratory distress syndrome (ARDS) or for Sepsis-Related Organ Failure Assessment (SOFA)[29,34]. Given both PaO_2 and FiO_2 as individual concepts, the PaO_2/FiO_2 ratio is provided by **ricu** as a recursive concept (`pafi`), requesting the two atomic concepts `pao2` and `fio2` and performing some form of imputation for when at a given time step one or both values are missing.

```
      "val_var": "heartrate",
      "class": "col_itm"
    }
  ],
  "mimic_demo": [
    {
      "ids": [211, 220045],
      "table": "chartevents",
      "sub_var": "itemid"
    }
  ]
}
```

Analogously to how different concept classes are used to represent different types of data, different item classes handle different data loading requirements. The most common scenario is selecting a subset of rows from a table by matching a set of ID values (`sub_itm`). In the above example, heart rate data in MIMIC-III can be located by searching for ID values 211 and 220045 in column `itemid` of table `chartevents` (heart rate data is stored in *long* format). Conversely, heart rate data in eICU is stored in *wide* format, requiring no row-subsetting. Column `heartrate` of table `vitalperiodic` contains all corresponding data and such data situations are handled by the `col_itm` class. Other item classes include `rgx_itm` where a regular expression is used for selecting rows and `fun_itm` where an arbitrary function can be used for data loading. If a data loading scenario is not covered by these classes, adding further `itm` subclasses is encouraged.

In order to extend the current concept library both to new datasets and new concepts, further JSON files can be incorporated by adding paths to their enclosing directories to `RICU_CONFIG_PATH`. Concepts with names that exist in files of the same name but with higher precedence are only used for their `sources` entries, such that `hr` for `new_dataset` can be specified as follows, while concepts with non-existing names are treated as new concepts.

```
"hr": {
  "sources": {
    "new_dataset": [
      {
        "ids": 6640,
        "table": "numericitems",
        "sub_var": "itemid"
      }
    ]
  }
}
```

Central to providing the required flexibility for loading of certain data concepts that require some specific preprocessing are callback functions that can be specified for several *item* types. Functions (with appropriate signatures), designated as callback functions, are invoked on individual data items, before concept-related preprocessing is applied. A common scenario for this is unit of measurement conversion: In MIMIC-III data for example, several `itemid` values correspond to temperature measurements, some of which refer to temperatures measured in degrees Celsius whereas others are used for measurements in degrees Fahrenheit. As the information encoding which measurement corresponds to which `itemid` values is no longer available during concept-related preprocessing, this is best resolved at the level of individual data items. Several function factories are available for generating callback functions and `convert_unit()` is intended for covering unit conversions[‖]. Data *items* corresponding to the `temp` concept for MIMIC-III are specified as

[‖]The presented implementation of this concept predates the addition of automatic unit conversion using the **units** package. While the concept definition as used by **ricu** will be updated to reflect these new capabilities, this example remains for illustration purposes.

```
{
  "mimic_demo": [
    {
      "ids": [676, 677, 223762],
      "table": "chartevents",
      "sub_var": "itemid"
    },
    {
      "ids": [678, 679, 223761, 224027],
      "table": "chartevents",
      "sub_var": "itemid",
      "callback": "convert_unit(fahr_to_cels, 'C', 'f')"
    }
  ]
}
```

indicating that for ID values 676, 677 and 223762 no preprocessing is required and for the remaining ID values the function `fahr_to_cels()` is applied to entries of the `val_var` column where the regular expression `"f"` is `TRUE` for the `unit_var` column (the values of which being ultimately replaced with `"C"`).
