# Supplementary material for: ricu: R’s interface to intensive care data
Source: Gigascience. 2023 Jun 15;12:giad041. doi: 10.1093/gigascience/giad041 (PMC10268223; doi:10.1093/gigascience/giad041)
Supplement: giad041_Supplemental_Files [file giad041_supplemental_files.zip › supplementD.pdf]

Supplement D: Implementation Details

Data classes

In order to represent tabular ICU data, **ricu** provides several classes, all inheriting from `data.table`. The most basic of which, `id_tbl`, marks one (or several) columns as `id_vars` which serve to define a grouping (i.e., identify patients or unit stays). Inheriting from `id_tbl`, `ts_tbl` is capable of representing grouped time series data. In addition to `id_var` column(s), a single column is marked as `index_var` and is required to hold a base R `difftime` vector. Furthermore, `ts_tbl` contains a scalar-valued `difftime` object as `interval` attribute, specifying the time series step size. More recently, a further class, `win_tbl`, inheriting from `ts_tbl` has been added. Objects of this class can be used for time-stamped measurements associated with a validity period. A set of drug infusions, consisting of both rates and intervals can as such be conveniently represented by a `win_tbl` object.

Metadata for classes inheriting from `id_tbl` is transiently added to `data.table` objects and for S3 generic functions which allow for object modifications, down-casting is implicit:

```
R> (dat <- ts_tbl(a = 1:5, b = hours(1:5), c = rnorm(5)))

# A `ts_tbl`: 5 x 3
# Id var:     `a`
# Index var:  `b` (1 hours)
      a b            c
  <int> <drtn>   <dbl>
1     1 1 hours  0.496
2     2 2 hours  0.564
3     3 3 hours -1.03
4     4 4 hours  0.818
5     5 5 hours  0.763

R> dat[["b"]] <- dat[["b"]] + mins(30)
R> dat

# An `id_tbl`: 5 x 3
# Id var:      `a`
      a b               c
  <int> <drtn>      <dbl>
1     1  5400 secs  0.496
2     2  9000 secs  0.564
3     3 12600 secs -1.03
4     4 16200 secs  0.818
5     5 19800 secs  0.763
```

Due to time series step size of `dat` being specified as 1 hour, an internal inconsistency is encountered when shifting time stamps by 30 minutes, as time steps are no longer multiples of the time series interval, in turn causing down-casting to `id_tbl`. Furthermore, if column `a` were to be removed, direct down-casting to `data.table` would be required in order to resolve resulting inconsistencies**.

**Updating an object inheriting from `id_tbl` using `data.table::set()` bypasses consistency checks as this is not an S3 generic function and therefore its behavior cannot be tailored to requirements of `id_tbl` objects. It therefore is up to the user to avoid creating invalid `id_tbl` objects in such a way.

Coercion to base classes `data.frame` and `data.table`, by stripping away the extra attributes, is easily possible using functions `as.data.frame()` and `as.data.table()`. Coercion is also available as `data.table`-style by-reference operation by passing `by_ref = TRUE` to any of the above coercion functions. User caution is advised, as this does break with base R by-value (or copy-on-modify) semantics and may lead to unexpected behavior.

In its current form, `win_tbl` objects can both be used to represent for example drug rates or drug amounts, administered over a specified time-period. When calling the utility function `expand()` however, which creates a `ts_tbl` from a `win_tbl` by assigning values to the corresponding time steps, values are assumed to be *valid* for the given interval.

```
R> (dat <- win_tbl(a = 1:5, b = hours(1:5), c = mins(rep(90, 5)),
+                  d = runif(5)))

# A `win_tbl`:  5 x 4
# Id var:       `a`
# Index var:    `b` (1 hours)
# Duration var: `c`
      a b       c           d
  <int> <drtn>  <drtn>  <dbl>
1     1 1 hours 90 mins 0.521
2     2 2 hours 90 mins 0.505
3     3 3 hours 90 mins 0.448
4     4 4 hours 90 mins 0.425
5     5 5 hours 90 mins 0.886

R> expand(dat)

# A `ts_tbl`: 10 x 3
# Id var:     `a`
# Index var:  `b` (1 hours)
       a b          d
   <int> <drtn>  <dbl>
 1     1 1 hours 0.521
 2     1 2 hours 0.521
 3     2 2 hours 0.505
 4     2 3 hours 0.505
 5     3 3 hours 0.448
 6     3 4 hours 0.448
 7     4 4 hours 0.425
 8     4 5 hours 0.425
 9     5 5 hours 0.886
10     5 6 hours 0.886
```

In a case where `d` represented drug amounts instead of drug rates, the current implementation of `expand()` would produce incorrect results. One would expect the overall amount in such a scenario to be evenly divided by – and the resulting fractions assigned to – the corresponding time steps. Allowing for this distinction is being considered, but, as of yet, has not been implemented.

Utilizing the attached metadata of objects inheriting from `id_tbl`, several utility functions can be called with concise semantics (as seen in the above example, where `expand()` is able to determine the required column names from the `win_tbl` object by default). Utilities include functions for sorting, checking for duplicates, aggregating data per combination of `id_vars` (and time step/time duration), checking time series data for gaps, verifying time series regularity and converting between irregular and regular time series, as well as

functions for several types of moving window operations. Adding to those class-specific implementations, `id_tbl` objects inherit from `data.table` (and therefore from `data.frame`), ensuring compatibility with a wide range of functionality targeted at these base-classes.

Data sources representation

Every dataset is represented by an environment with class attributes and associated metadata objects stored as object attributes to that environment. Dataset environments all inherit from `src_env` and from any number of class names constructed from data source name(s) with a suffix `_env` attached. The environment representing MIMIC-III, for example inherits from `src_env` and `mimic_env`, while the corresponding demo dataset inherits from `src_env`, `mimic_env` and `mimic_demo_env`. These sub-classes are later used for tailoring the process of data loading to particularities of individual datasets.

A `src_env` contains an active binding per associated table, which returns a `src_tbl` object representing the requested table. As is the case for `src_env` objects, `src_tbl` objects inherit from additional classes for reasons explained above. The `admissions` table of the MIMIC-III demo dataset for example, inherits from `mimic_demo_tbl` and `mimic_tbl` (alongside classes `src_tbl` and `prt`).

```
R> mimic_demo$admissions

# <mimic_tbl>: [129 x 19]
# ID options:  subject_id (patient) < hadm_id (hadm) < icustay_id (icustay)
# Defaults:    `admission_type` (val)
# Time vars:   `admittime`, `dischtime`, `deathtime`, `edregtime`, `edouttime`
    row_id subjec~ hadm_id admittime           dischtime
     <int>   <int>   <int> <dttm>              <dttm>
  1  12258   10006  142345 2164-10-23 21:09:00 2164-11-01 17:15:00
  2  12263   10011  105331 2126-08-14 22:32:00 2126-08-28 18:59:00
  3  12265   10013  165520 2125-10-04 23:36:00 2125-10-07 15:13:00
  4  12269   10017  199207 2149-05-26 17:19:00 2149-06-03 18:42:00
  5  12270   10019  177759 2163-05-14 20:43:00 2163-05-15 12:00:00
...
125  41055   44083  198330 2112-05-28 15:45:00 2112-06-07 16:50:00
126  41070   44154  174245 2178-05-14 20:29:00 2178-05-15 09:45:00
127  41087   44212  163189 2123-11-24 14:14:00 2123-12-30 14:31:00
128  41090   44222  192189 2180-07-19 06:55:00 2180-07-20 13:00:00
129  41092   44228  103379 2170-12-15 03:14:00 2170-12-24 18:00:00
# ... with 119 more rows, and 14 more variables: deathtime <dttm>,
#   admission_type <chr>, admission_location <chr>, discharge_location <chr>,
#   insurance <chr>, language <chr>, religion <chr>, marital_status <chr>,
#   ethnicity <chr>, edregtime <dttm>, edouttime <dttm>, diagnosis <chr>,
#   hospital_expire_flag <int>, has_chartevents_data <int>
```

Powered by the **prt**[35] package, `src_tbl` objects represent row-partitioned tabular data stored as multiple binary files created by the **fst**[36] package. In addition to standard subsetting, `prt` objects can be subsetted via the base R S3 generic function `subset()` and using non-standard evaluation (NSE):

```
R> subset(mimic_demo$admissions, subject_id > 44000, language:ethnicity)

   language          religion marital_status              ethnicity
1:     ENGL          CATHOLIC         SINGLE                  WHITE
2:     ENGL          CATHOLIC         SINGLE                  WHITE
3:     ENGL          CATHOLIC         SINGLE                  WHITE
```

```
4:     ENGL PROTESTANT QUAKER        MARRIED                  WHITE
5:     ENGL      UNOBTAINABLE         SINGLE BLACK/AFRICAN AMERICAN
6:     ENGL          CATHOLIC         SINGLE                  WHITE
7:     ENGL     NOT SPECIFIED         SINGLE                  WHITE
```

This syntax makes it possible to read row-subsets of *long* tables into memory with little memory overhead. While terseness of such an API does introduce potential ambiguity, this is mostly overcome by using the tidy eval framework provided by **rlang**[37]:

```
R> subject_id <- 44000:45000
R> subset(mimic_demo$admissions, .data$subject_id %in% .env$subject_id,
+         subject_id:dischtime)

   subject_id hadm_id           admittime           dischtime
1:      44083  125157 2112-05-04 08:00:00 2112-05-11 14:15:00
2:      44083  131048 2112-05-22 15:37:00 2112-05-25 13:30:00
3:      44083  198330 2112-05-28 15:45:00 2112-06-07 16:50:00
4:      44154  174245 2178-05-14 20:29:00 2178-05-15 09:45:00
5:      44212  163189 2123-11-24 14:14:00 2123-12-30 14:31:00
6:      44222  192189 2180-07-19 06:55:00 2180-07-20 13:00:00
7:      44228  103379 2170-12-15 03:14:00 2170-12-24 18:00:00
```

By using **rlang** pronouns (`.data` and `.env`), the distinction can readily be made between a name referring to an object within the context of the data and an object within the context of the calling environment.

Data source setup

In order to make a dataset accessible to **ricu**, three steps are necessary, each handled by an exported S3 generic function: `download_src()`, `import_src()` and `attach_src()`. The first two steps, data download and import, are one-time procedures, whereas attaching is carried out every time the package namespace is loaded. By default, all data sources known to **ricu** are configured to be attached and in case some data is missing for a given data source, the missing data is downloaded and imported on first access. An outline of the steps involved for data source setup is shown in Figure 3.

Data download

The first step towards accessing data is data download, taken care of by the S3 generic function `download_src()`. For the datasets included with **ricu**, prior to calling `download_src()`, the following environment variables can be set (indicated in red in the $a \to b$ edge in Figure 3):

- `RICU_PHYSIONET_USER`/`RICU_PHYSIONET_PASS`: PhysioNet login credentials with access to the requested dataset(s).
- `RICU_AUMC_TOKEN`: Download token, extracted from the download URL received after being granted data access.

If any of the required access credentials are not available as environment variables, they can be supplied as function arguments to `download_src()` or the user is queried in interactive sessions and an error is thrown otherwise.

As a quick reminder on system requirements for initial data setup operations: Each of the supported datasets requires 5-10 GB disk space for permanent storage and 50-100 GB of temporary disk storage during download and import. Memory requirements are kept low (8-16 GB) by performing all setup operations only on subsets of rows at the time. Initial data source setup can be expected to take upwards of an hour per dataset.

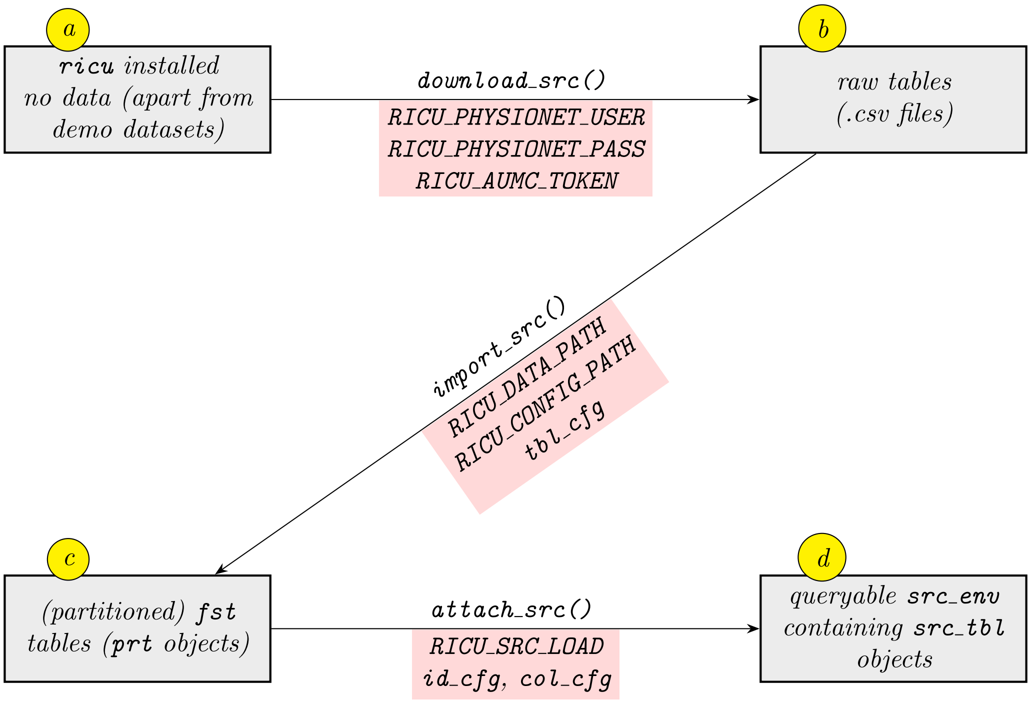

Figure 3. Making a dataset available to **ricu** involves several steps, starting with data download, followed by preparation for efficient access and finalized by instantiation of data structures containing relevant metadata. The functions which are used for each step are displayed above arrows and below (in red) are indicated specific configuration settings or environment variables which are need for (or can be used to customize) the specific step.

Data import

After successful data download, importing prepares tables for efficient random row- and column-access, for which the raw data format (.csv) is not well suited (see edge $b \rightarrow c$ in Figure 3). Tables are read in using **readr**[38], potentially (re-)partitioned row-wise, and re-saved using **fst**. Environment variables that can be set to customize **ricu** data handling, relevant for import and attaching include:

- `RICU_DATA_PATH`: Optional data storage location (if unset, this defaults to a system-specific, user-specific directory). The current value used for this setting can be queried by calling `data_dir()`.
- `RICU_CONFIG_PATH`: A comma-separated set of paths to directories containing configuration files. The current set of paths is retrievable by calling `config_paths()` and the ordering of paths determines precedence of how configuration files are combined (if multiple files of the same name are available).

For importing, the information contained in `tbl_cfg` configuration objects is most relevant. This determines column data types, table partitioning and sanity checks like number of rows per table. Please refer to Table configuration section for more information on the construction of `tbl_cfg` objects.

Data attaching

Finally, attaching a dataset creates a corresponding `src_env` object, containing a corresponding `src_tbl` object for each table, which together with associated metadata are used by **ricu** to run queries against the data (edge $c \rightarrow d$ in Figure 3). The environment variable `RICU_SRC_LOAD` may contain a comma-separated list of data source names that are set up for being automatically attached on namespace loading. This defaults to all currently supported datasets and the active set of source names is available as `auto_attach_srcs()`. Apart from this automatism, the process of attaching a dataset can be manually invoked by calling `attach_src()`, which can be convenient when for example updating the data source configuration after it has been modified.

Two configuration objects which are important for data loading (see the following Data loading section) are `id_cfg` and `col_cfg` (described in ID configuration and Default column configuration sections, respectively), providing default values for certain types of columns, including time-stamp, measurement value and measurement unit column names, as well as defining relationships between patient identifiers (such as hospital stay ID and ICU stay ID).

Data loading

The lowest level of data access is direct subsetting of `src_tbl` objects as shown at the start of Data sources representation section. As `src_tbl` inherits from `prt`, the `subset()` implementation provided by **prt** can be used for NSE of data-expressions against on-disk, tabular data. Building on that, several S3 generic functions successively homogenize data representations as visualized in Figure 4.

The most basic layer in data loading is provided by the S3 generic function `load_src()`, which provides a string-based interface to the `cols` argument of `subset()` while forwarding the unevaluated expression passed as `rows` (see edge $a \rightarrow b$ in Figure 4).

```
R> load_src(mimic_demo$admissions, subject_id > 44000,
+           cols = c("hadm_id", "admittime", "dischtime"))

   hadm_id           admittime           dischtime
1:  125157 2112-05-04 08:00:00 2112-05-11 14:15:00
2:  131048 2112-05-22 15:37:00 2112-05-25 13:30:00
3:  198330 2112-05-28 15:45:00 2112-06-07 16:50:00
4:  174245 2178-05-14 20:29:00 2178-05-15 09:45:00
5:  163189 2123-11-24 14:14:00 2123-12-30 14:31:00
```

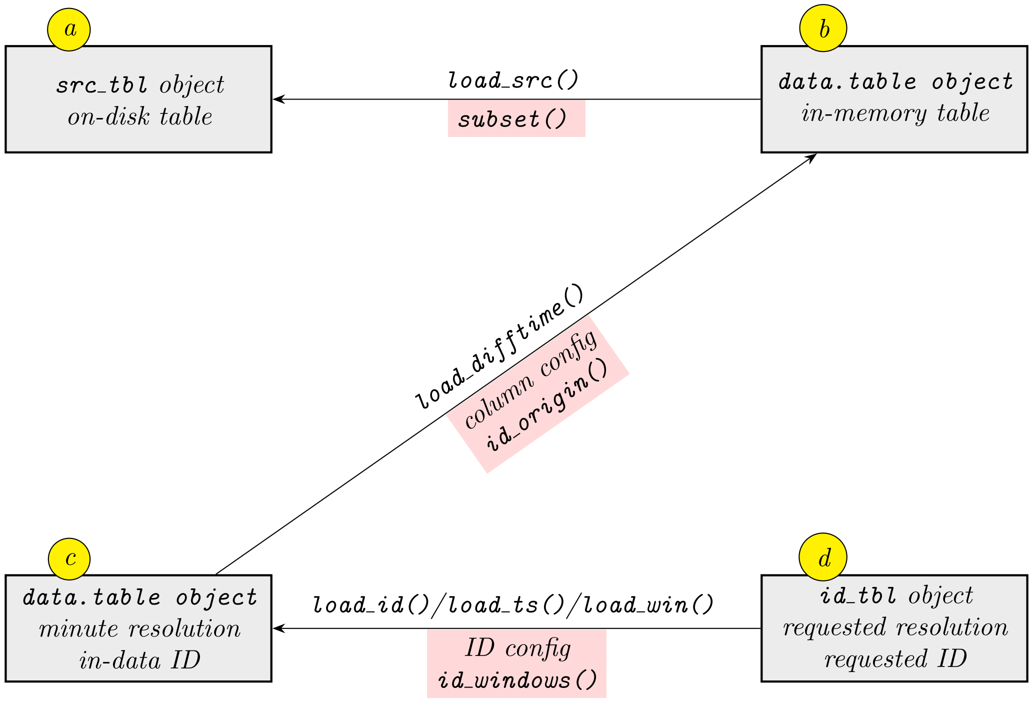

Figure 4. Data loading proceeds through several layers, each contributing a step towards harmonizing discrepancies among raw data representations provided by the different data sources. Raw data tables are represented by **ricu** as `src_tbl` objects which can be queried using `load_src()`. Absolute time-stamps in the returned `data.table` are converted to times relative to admission (in minutes) by `load_difftime()` and finally, `load_id()`/`load_ts()`/`load_win()` ensure a given ID system and time interval.

```
6:  192189 2180-07-19 06:55:00 2180-07-20 13:00:00
7:  103379 2170-12-15 03:14:00 2170-12-24 18:00:00
```

As data sources differ in their representation of time-stamps, a next step in data homogenization is to converge to a common format: the time difference to the origin time-point of a given ID system (for example ICU admission).

```
R> load_difftime(mimic_demo$admissions, subject_id > 44000,
+               cols = c("hadm_id", "admittime", "dischtime"))

# An `id_tbl`: 7 x 3
# Id var:      `hadm_id`
  hadm_id admittime dischtime
    <int> <drtn>    <drtn>
1  103379 0 mins    13846 mins
2  125157 0 mins    10455 mins
3  131048 0 mins     4193 mins
4  163189 0 mins    51857 mins
5  174245 0 mins      796 mins
6  192189 0 mins     1805 mins
7  198330 0 mins    14465 mins
```

The function `load_difftime()` is expected to return timestamps as base R `difftime` vectors (in minutes; edge $b \rightarrow c$ in Figure 4). The argument `id_hint` can be used to specify a preferred ID system, but if not available in raw data, `load_difftime()` will return data using the ID system with highest cardinality (i.e., ICU stay ID is preferred over hospital stay ID). In the above example, if `icustay_id` were requested, data would be returned using `hadm_id`, whereas a `subject_id` request would be honored, as the corresponding ID column is available in the `admissions` table.

Building on `load_difftime()` functionality, functions `load_id()`/`load_ts()`/`load_win()` return `id_tbl`/`ts_tbl`/`win_tbl` objects with the requested ID system (passed as `id_var` argument). This uses raw data IDs if available or calls `change_id()` in order to convert to the desired ID system (edge $c \rightarrow d$ in Figure 4). Similarly, where `load_difftime()` returns data with fixed time interval of one minute, `load_id()` allows for arbitrary time intervals (using `change_interval()`; defaults to 1 hour).

```
R> load_id(mimic_demo$admissions, subject_id > 44000,
+         cols = c("admittime", "dischtime"), id_var = "hadm_id")

# An `id_tbl`: 7 x 3
# Id var:      `hadm_id`
  hadm_id admittime dischtime
    <int> <drtn>    <drtn>
1  103379 0 hours   230 hours
2  125157 0 hours   174 hours
3  131048 0 hours    69 hours
4  163189 0 hours   864 hours
5  174245 0 hours    13 hours
6  192189 0 hours    30 hours
7  198330 0 hours   241 hours
```

Throughout several of theses functions, `col_cfg` objects are used to provide sensible defaults. In order to convert to relative times, `load_difftime()`, for example, requires names of columns for which this applies (provided by the `time_vars` entry), and `load_ts()` needs to know which of the `time_vars` to use as

`index_var`. For more information on the construction of `col_cfg` objects, please refer to the Default column configuration section.

A call to `change_id()` requires the construction of a table which contains the mapping between different ID systems, together with information about how to convert timestamps between these ID systems (edge $c \to d$ in Figure 4). The function responsible for providing the necessary information is `id_windows()` and the associated S3 generic function `id_win_helper()`. The entry point `id_windows()` wraps `id_win_helper()`, providing memoization, as the resulting structure is expensive to compute relative to the frequency of being required.

```
R> id_windows(mimic_demo)

# An `id_tbl`: 136 x 9
# Id var:      `icustay_id`
    icustay_id hadm_id subject~ icustay~ hadm_i~ subjec~ icusta~ hadm_i~ subjec~
         <int>   <int>    <int> <drtn>   <drtn>  <drtn>  <drtn>  <drtn>  <drtn>
  1     201006  198503    10076 0 mins    -3290~ -36222~ 10249 ~  9114 ~    839~
  2     201204  114648    42321 0 mins       -2~ -42371~  2753 ~  6949 ~  11826~
  3     203766  126949    10045 0 mins    -1336~ -36116~  9077 ~  8818 ~    871~
  4     204132  157609    40310 0 mins       -1~ -21593~ 10366 ~ 10103 ~    910~
  5     204201  177678    10104 0 mins     -368~ -37090~   954 ~  9445 ~ 247393~
...
132     295043  170883    10124 0 mins   -10413~ -43835~  4170 ~ 31258 ~   3009~
133     295741  176805    10090 0 mins       -1~ -14660~  1643 ~  3153 ~ 117273~
134     296804  110244    10035 0 mins    -1294~ -39914~  1981 ~  4599 ~ 214046~
135     297782  167612    43909 0 mins       -1~ -41487~   274 ~   207 ~   -114~
136     298685  151323    42075 0 mins       -1~ -42087~ 19234 ~ 19082 ~   1908~
# ... with 126 more rows
```

Analogously, the function pair `id_origin()` and `id_orig_helper()`, with the former wrapping the latter and again providing memoization, is used for datasets where time-stamps are represented by absolute times, returning the origin time-points for a given ID system which then can be used to calculate relative times (edge $b \to c$ in Figure 4).

```
R> id_origin(mimic_demo, "icustay_id")

# An `id_tbl`: 136 x 2
# Id var:      `icustay_id`
    icustay_id intime
         <int> <dttm>
  1     201006 2107-03-24 04:06:14
  2     201204 2121-12-07 20:50:36
  3     203766 2129-11-24 22:46:57
  4     204132 2144-12-24 16:16:41
  5     204201 2120-08-24 23:47:23
...
132     295043 2192-04-24 02:29:49
133     295741 2124-01-12 14:27:16
134     296804 2129-03-04 13:40:11
135     297782 2152-10-09 19:05:36
136     298685 2166-02-12 17:57:37
# ... with 126 more rows
```

For the included datasets, the implementations of `id_win_helper()` and `id_orig_helper()`, use information contained in `id_cfg` objects (see ID configuration section) to determine which columns in which tables are required for constructing the corresponding lookup tables. Doing so, however, is not necessary: an `id_win_helper()` implementation for a new dataset could forego this by hard-coding table/column names as part of the function logic, in-turn simplifying the corresponding `id_cfg` object to merely providing naming and ordering information.

Data source configuration

Data source environments (and corresponding `src_tbl` objects) are constructed using source configuration objects: list-based structures, inheriting from `src_cfg` and from any number of data source specific class names with suffix `_cfg` appended (as discussed at the beginning of Data sources representation section). The exported function `load_src_cfg()` reads a JSON formatted file and creates a `src_cfg` object per data source and further therein contained objects.

```
R> cfg <- load_src_cfg("mimic_demo")
R> str(cfg, max.level = 3L, width = 70L)

List of 1
 $ mimic_demo:List of 6
  ..$ name   : chr "mimic_demo"
  ..$ prefix : chr [1:2] "mimic_demo" "mimic"
  ..$ id_cfg : id_cfg [1:3] `subject_id`, `hadm_id`, `icustay_id`
  ..$ col_cfg: col_cfg [1:25] [0, 0, 5, 0, 1], [0, 1, 6, 0, 1], [1, 0, 0...
  ..$ tbl_cfg: tbl_cfg [1:25] [?? x 19; 1], [?? x 24; 1], [?? x 4; 1], [...
  ..$ extra  :List of 1
  .. ..$ url: chr "https://physionet.org/files/mimiciii-demo/1.4"
  ..- attr(*, "class")= chr [1:3] "mimic_demo_cfg" "mimic_cfg" "src_cfg"

R> mi_cfg <- cfg[["mimic_demo"]]
```

In addition to required fields `name` and `prefix` (used as class prefix), as well as further arbitrary fields contained in `extra` (`url` in this case), several configuration objects are part of `src_cfg`: `id_cfg`, `col_cfg` and `tbl_cfg`.

ID configuration

An `id_cfg` object contains an ordered set of key-value pairs representing patient identifiers in a dataset. An implicit assumption currently is that a given patient ID system is used consistently throughout a dataset, meaning that for example an ICU stay ID is always referred to by the same name throughout all tables containing a corresponding column. Owing to the relational origins of these datasets this has been fulfilled in all instances encountered so far. In MIMIC-III, ID systems

```
R> as_id_cfg(mi_cfg)

<id_cfg<mimic_demo[patient < hadm < icustay]>[3]>
     patient          hadm      icustay
`subject_id`     `hadm_id` `icustay_id`
```

are available, allowing for identification of individual patients, their (potentially multiple) hospital admissions over the course of the years and their corresponding ICU admissions (as well as potential re-admissions). Ordering corresponds to cardinality: moving to larger values implies moving along a one-to-many relationship. This information is used in data-loading, whenever the target ID system is not contained in the raw data.

Default column configuration

Again used in data loading, this per-table set of key-value pairs specifies column defaults as `col_cfg` object. Each key describes a type of column with special meaning and the corresponding value specifies said column for a given table. The print method for `col_cfg` reports all keys alongside the per-table counts of accordingly registered values (i.e., columns).

```
R> as_col_cfg(mi_cfg)
```

```
<col_cfg<mimic_demo[id, index, time, unit, val]>[25]>
        admissions            callout         caregivers        chartevents
   [0, 0, 5, 0, 1]    [0, 1, 6, 0, 1]    [1, 0, 0, 0, 1]    [0, 1, 2, 1, 1]
         cptevents              d_cpt    d_icd_diagnoses   d_icd_procedures
   [0, 1, 1, 0, 1]    [1, 0, 0, 0, 1]    [1, 0, 0, 0, 1]    [1, 0, 0, 0, 1]
           d_items         d_labitems     datetimeevents      diagnoses_icd
   [1, 0, 0, 0, 1]    [1, 0, 0, 0, 1]    [0, 1, 3, 0, 1]    [0, 0, 0, 0, 1]
          drgcodes           icustays     inputevents_cv     inputevents_mv
   [0, 0, 0, 0, 1]    [0, 1, 2, 0, 1]    [0, 1, 2, 1, 1]    [0, 1, 4, 1, 1]
         labevents microbiologyevents       outputevents           patients
   [0, 1, 1, 1, 1]    [0, 1, 2, 0, 1]    [0, 1, 2, 1, 1]    [0, 0, 4, 0, 1]
     prescriptions procedureevents_mv     procedures_icd           services
   [0, 1, 2, 1, 1]    [0, 1, 4, 1, 1]    [0, 0, 0, 0, 1]    [0, 1, 1, 0, 1]
         transfers
   [0, 1, 2, 0, 1]
```

The following column defaults are currently in use throughout **ricu** but the set of keys can be extended to arbitrary new values:

- `id_var`: In case a table does not contain at least one ID column corresponding to one of the ID systems specified as `id_cfg`, the default ID column can be set on a per-table basis as `id_var`[††].
- `index_var`: A column that is used to define an ordering in time over rows, thereby providing a time series index[‡‡].
- `time_vars`: Columns which will be treated as time variables (important for converting between ID systems for example), but not as time series indices[§§].
- `unit_var`: Used in concept loading (more specifically for `num_cncpt` concepts, see Concept specification section) to identify columns that represent unit of measurement information.
- `val_var`: Again used when loading data concepts, this identified a default value variable in a table, representing the column of interest to be used as returned data column.

While `id_var`, `index_var` and `time_vars` are used to provide sensible defaults to functions used for general data loading (Data loading section), `unit_var`, `val_var`, as well as potential user-defined defaults are only used in concept loading (see Ready-to-use concepts section) and therefore need not be prioritized when integrating new data sources until data concepts have been mapped.

[††]This for example is the case for the `d_items` table in MIMIC-III, which does not contain any patient related data, but holds information on items encoding types of measurements, procedures, etc., used throughout other tables holding actual patient data.

[‡‡]For the MIMIC-III table `inputevents_mv`, of the four available time variables (`starttime`, `endtime`, `storetime`, `comments_date`), `starttime` lends itself to be used as index variable more than the other candidates and therefore is set as default.

[§§]In case of the `admissions` table in MIMIC-III for example, a total of five columns are considered to be time variables, none of which stands out as potential `index_var`.

Table configuration

Finally, `tbl_cfg` objects are used during the initial setup of a data source. In order to create a representation of a table that is accessible by **ricu** from raw data, several key pieces of information are required:

- File name(s): In the simplest case, a single file corresponds to a single table. Other scenarios that have been encountered (and are therefore handled) include tables partitioned into multiple files and .tar archives containing multiple tables.
- Column specification: For each column, the expected data type has to be known, as well as a pair of names, one corresponding to the raw data column name and one corresponding to the column name to be used within **ricu**.
- (Optional) number of rows: Used as sanity check whenever available.
- (Optional) partitioning information: For very *long* tables it can be useful to specify a row-partitioning. This currently is only possible by applying a vector of breakpoints to a single numeric column, thereby defining a grouping.

Table configuration objects are only used within the context of the functions `download_src()` and `import_src()` and are therefore not required if download and import are carried out manually.

```
R> as_tbl_cfg(mi_cfg)
```

```
<tbl_cfg<mimic_demo[rows x cols; partitions]>[25]>
        admissions           callout         caregivers        chartevents
       [?? x 19; 1]       [?? x 24; 1]        [?? x 4; 1]       [?? x 15; 2]
         cptevents             d_cpt    d_icd_diagnoses   d_icd_procedures
       [?? x 12; 1]        [?? x 9; 1]        [?? x 4; 1]        [?? x 4; 1]
           d_items        d_labitems     datetimeevents      diagnoses_icd
       [?? x 10; 1]        [?? x 6; 1]       [?? x 14; 1]        [?? x 5; 1]
          drgcodes          icustays     inputevents_cv     inputevents_mv
        [?? x 8; 1]       [?? x 12; 1]       [?? x 22; 1]       [?? x 31; 1]
         labevents microbiologyevents      outputevents           patients
        [?? x 9; 1]       [?? x 16; 1]       [?? x 13; 1]        [?? x 8; 1]
     prescriptions procedureevents_mv    procedures_icd           services
       [?? x 19; 1]       [?? x 25; 1]        [?? x 5; 1]        [?? x 6; 1]
         transfers
       [?? x 13; 1]
```

For the `chartevents` table of the MIMIC-III demo dataset, rows are partitioned into two groups, while all other tables are represented by a single partition. Furthermore, the expected number of rows is unknown (`??`) as this is missing from the corresponding `tbl_cfg` object.

Adding external datasets

In order to add a new dataset to **ricu**, several aspects outlined in the previous subsections require consideration. For illustration purposes, code for integrating AmsterdamUMCdb as external dataset is available from GitHub. While this is no longer needed for using the `aumc` data source, the repository will remain as it might serve as template to integration of new datasets. Throughout this repository (and the following paragraphs), the AmsterdamUMCdb data treated as an **ricu**-external dataset is referred to as `aumc_ext`.

Adding configuration information

Central to adding a new dataset to **ricu** is providing some configuration information in a `data-sources.json` file pointed to by the environment variable `RICU_CONFIG_PATH`. Depending on particularities of the dataset in question, corresponding implementations of some of the S3 generic functions mentioned throughout Data source setup and Data loading sections might have to be provided. The amount of confirmation information required to get started also depends on the desired level of integration. As data download and import are one-time procedures, these steps can be carried out manually, negating the need for specifying column data types in `data-sources.json` and providing data source specific methods for the `download_src()` and `import_src()` generics.

The basic organization of a data source configuration entry, as it could be used for `aumc_ext`, specified as JSON is as follows:

```
{
  "name": "aumc_ext",
  "id_cfg": {
    "patient": {
      "id": "patientid",
      "position": 1
    },
    "icustay": {
      "id": "admissionid",
      "position": 2
    }
  },
  "tables": {
    ...
  }
}
```

The shown `id_cfg` entry represents the minimally required set of entries, where for each ID specification, `start`, `end` and `table` are omitted (when compared to the `aumc` configuration provided by **ricu**). The `tables` entry expands to something like the following:

```
"tables": {
  "freetextitems": {
  },
  "drugitems": {
    "defaults": {
      "index_var": "start",
      "val_var": "dose",
      "unit_var": "doseunit",
      "time_vars": ["start", "stop"]
    }
  },
  "numericitems": {
    "defaults": {
      "index_var": "measuredat",
      "val_var": "value",
      "unit_var": "unit",
      "time_vars": ["measuredat", "registeredat", "updatedat"]
    },
    "partitioning": {
```

```
      "col": "",
      "breaks": [
        0, 0, 0, 0, 0, 0, 0, 0, 0, 0, 0, 0, 0, 0, 0, 0, 0, 0,
        0, 0, 0, 0, 0
      ]
    }
  },
  ...
}
```

Minimally required is simply an entry indicating the data source membership of a table (if not partitioned; cf., `freetextitems`). This does slightly complicate data exploration, as if no `defaults` are available, no default values can be provided to calls to `load_ts()` and related functions and therefore repeatedly have to be specified in corresponding function calls. Also, when specifying data items in such a setup, the per-table column names for special columns such as `index_var`, `val_var`, etc., have to be repeated for each individual item entry.

For partitioned tables, the basic structure of a `partitioning` entry is required, but the content itself is irrelevant, as this is only used for setup (cf., `numericitems`). The length of `breaks`, however, is required to match the number of partitions (i.e., a length 23 `breaks` specification corresponds to a partitioning into 24 row-groups.)[¶¶]. The directory containing such a `data-sources.json` can then be pointed to by the environment variable `RICU_CONFIG_PATH`, making it available to **ricu**.

Enabling data loading

As for functions that are required, currently there is no default method available for the loading step provided by `load_difftime()` and most likely an implementation of the generic function `id_win_helper()` will be required as well. For `aumc_ext`, `load_difftime()` could be implemented as

```
R> ms_as_min <- function(x) {
+   as.difftime(as.integer(x / 6e4), units = "mins")
+ }
R>
R> aumc_difftime <- function(x, rows, cols = colnames(x),
+                            id_hint = id_vars(x),
+                            time_vars = ricu::time_vars(x), ...) {
+
+   if (id_hint %in% colnames(x)) {
+     id_sel <- id_hint
+   } else {
+     id_opt <- id_var_opts(sort(as_id_cfg(x), decreasing = TRUE))
+     id_sel <- intersect(id_opt, colnames(x))[1L]
+   }
+
+   stopifnot(is.character(id_sel), length(id_sel) == 1L)
+
+   if (!id_sel %in% cols) {
+     cols <- c(id_sel, cols)
+   }
+
+   time_vars <- intersect(time_vars, cols)
```

[¶¶]Originally it was intended to use partitioning information during data loading in order to narrow down the set of partitions that have to be accessed. So far, this optimization has not been implemented.

```
+
+   dat <- load_src(x, {{ rows }}, cols)
+   dat <- dat[, c(time_vars) := lapply(.SD, ms_as_min),
+              .SDcols = time_vars]
+
+   as_id_tbl(dat, id_vars = id_sel, by_ref = TRUE)
+ }
```

Such a function attempts to use the ID as requested as `id_hint`, but falls back to the best possible alternative (using the ordering as previously specified in the `id_cfg` JSON configuration) if not provided by the data. The helper function `id_var_opts()` returns the dataset-specific column names of an `id_cfg` object (as opposed to the dataset-agnostic ID names; cf., `subject_id` and `patient`). Both the row-subsetting expression and column selection are passed on to `load_src()` and all columns specified as `time_vars` are converted to `difftime` vectors in minutes. Operations can safely be carried out using by-reference semantics, as intermediate objects are not exposed to the user.

For a possible implementation of the `id_win_helper()` generic, column and table names to assemble the desired lookup table are hard coded instead of provided by the corresponding `id_cfg` object (as is the case in the **ricu**-internal implementation).

```
R> aumc_windows <- function(x) {
+
+   ids <- c("admissionid", "patientid")
+   sta <- c("admittedat", "firstadmittedat")
+   end <- c("dischargedat", "dateofdeath")
+
+   tbl <- as_src_tbl(x, "admissions")
+
+   res <- tbl[, c(ids, sta[1L], end)]
+   res <- res[, c(sta[2L]) := 0L]
+   res <- res[, c(sta, end) := lapply(.SD, ms_as_min),
+              .SDcols = c(sta, end)]
+
+   res <- data.table::setcolorder(res, c(ids, sta, end))
+   res <- rename_cols(res, c(ids, paste0(ids, "_start"),
+                                  paste0(ids, "_end")), by_ref = TRUE)
+
+   as_id_tbl(res, ids[2L], by_ref = TRUE)
+ }
```

As all the required information is available form the `admissions` table, `aumc_windows()` simply loads the corresponding columns, converts them to minute resolution, followed by some renaming. ICU admissions and discharges in this table are relative to initial hospital admissions and therefore an all-zero column `firstadmittedat` is added and the `id_var` of the resulting `id_tbl` is marked as `patientid`[***].

A final step in making a new dataset accessible to **ricu** lies in specifying concept items. To this end, a file `concept-dict.json` can be added to the directory pointed to by the environment variable `RICU_CONFIG_PATH`, containing entries like the following, which will make it possible to use the `hr` concept across all datasets included with **ricu**, alongside the newly added dataset.

[***]The patient ID created in this way is different to that available for MIMIC-III, where patient date of birth is provided. An approximate date of birth could be constructed if ages were reported more precisely, but given the rough binning available here, this might be considered an acceptable limitation of resulting patient IDs. Nevertheless awareness of such differences in data presentation is important.

```
{
  "hr": {
    "sources": {
      "aumc_ext": [
        {
          "ids": 6640,
          "table": "numericitems",
          "sub_var": "itemid"
        }
      ]
    }
  }
}
```

The above outline serves as an example on how to proceed when adding new data to **ricu**. Aspects like having multiple patient IDs, for example, could be further simplified[†††]. Owing to the extensive use of S3 generic functions, **ricu** offers considerable flexibility for customizing certain behavior to specifics of a given data source, while providing fallback procedures whenever more general treatment can be applied.

Summary of required steps

Summarizing aspects explained in more detail in the previous sections, the following points list the required steps for adding new data in the order they should be considered in. The approach taken here being is to start simple and expand.

1. Tables saved as `.fst` files should be moved to the folder returned by `src_data_dir()` when passed the dataset name (alternatively, methods implementing `src_download()` and `src_import()` are required).

2. A minimal data source configuration file `data-sources.json` is required in the directory pointed to by `RICU_CONFIG_PATH`. For AmsterdamUMCdb, this could be as minimal as (assuming no partitioning):

   ```
   {
     "name": "aumc_min",
     "id_cfg": {
       "icustay": "admissionid"
     },
     "tables": {
       "admissions": {},
       "drugitems": {},
       "freetextitems": {},
       "listitems": {},
       "numericitems": {},
       "procedureorderitems": {},
       "processitems": {}
     }
   }
   ```

   File names have to match table names, i.e., the admissions table should be named `admissions.fst`. Upon a call to `attach_src()` (or next loading of the package and having added the data source name to `RICU_SRC_LOAD`) the new data source can be explored using `load_src()`.

[†††] An example for such a reduced setup is available from the AUMC GitHub repository as `aumc_min`. Moving to only a single patient identifier also does away with the need for a `id_win_helper()` implementation, as `change_id()` will not be called in such a scenario.

3. A `load_difftime()` method is required, which:

   - passes a row-subsetting expression to `load_src()` using the **rlang** curly-curly operator,
   - converts columns passed as `time_vars` to minute-resolution `difftime` vectors,
   - returns an `id_tbl` object where patient identifiers are chosen such that time-stamps are relative to corresponding admission,
   - (optionally) uses the column passed as `id_hint` for patient identifiers, if multiple identifiers are available from data.

   Upon registering this method with S3 dispatch, higher-level data loading functions such as `load_ts()` become available (given that no changes in patient identifiers are requested).

4. (Optional) if the source configuration specifies multiple patient identifiers which are not all available from all tables directly, an implementation of `id_win_helper()` most likely will be required (see Data loading section).

5. Now, the source configuration can be expanded with per-table column defaults and data items can be added to the concepts included with **ricu** by creating a `concept-dict.json` under the path pointed to by `RICU_CONFIG_PATH`. For more information on readily available concepts, refer to the Ready-to-use concepts section and for specifying new concepts altogether, pointers are available in section Concept specification.
