# Supplementary material for: ricu: R’s interface to intensive care data
Source: Gigascience. 2023 Jun 15;12:giad041. doi: 10.1093/gigascience/giad041 (PMC10268223; doi:10.1093/gigascience/giad041)
Supplement: giad041_Reviewer_1_Report_Original_Submission — Chris Armit -- 2/7/2023 Reviewed [file giad041_reviewer_1_report_original_submission.pdf]

Reviewer Report

Title: ricu: R's Interface to Intensive Care Data

Version: Original Submission **Date:** 2/7/2023

Reviewer name: Chris Armit

Reviewer Comments to Author:

This Technical Note describes the software tool Ricu, which is "R's Interface to Intensive Care Data". The core objective of this software tool and its accompanying study is "to develop a unified framework for analyzing data from five large, publicly available ICU datasets." The manuscript is well written, and the code for Ricu is made publicly available from GitHub (https://github.com/eth-mds/ricu). Importantly, this repository has been ascribed an OSI-approved GPL-3.0 license which encourages reuse. An alternative CRAN link is also provided in the manuscript (https://CRAN.R-project.org/package=ricu).For the supporting data, AmsterdamUMCdb is Restricted Access and reviewers must first complete the Data or Specimens Only Research (DSOR) course from CITI. Likewise, the complete MIMIC-III, eICU, HiRID and MIMIC-IV datasets can be accessed by registering and setting up a credentialed account at PhysioNet (https://physionet.org/). Only credentialed users who sign the Data Use Agreement can access the files.Minor commentI initially encountered difficulty in running Ricu using RStudio. I used the MIMIC-III and eICU demo datasets to test the Ricu software tool, and I was unable to run the command explain_dictionary(). However, the troubleshooting guidelines that the authors provided were very helpful and I am now able to run Ricu on macOS Ventura. The troubleshooting guidelines are below:***You just need to runlibrary(ricu)before running the explain_dictionary() function. Also, note that the line> remotes::install_github("eth-mds/ricu")resulted in an error due to the remotes package not being installed (to fix this, you can just run install.packages("remotes"), or alternatively use the ricu version from CRAN which is installed with install.packages("ricu")).***I request that the authors add these troubleshooting details to the Ricu documentation.

Level of Interest

Please indicate how interesting you found the manuscript: Choose an item.

Quality of Written English

Please indicate the quality of language in the manuscript: Choose an item.

Declaration of Competing Interests

Please complete a declaration of competing interests, considering the following questions:

- Have you in the past five years received reimbursements, fees, funding, or salary from an organisation that may in any way gain or lose financially from the publication of this manuscript, either now or in the future?
- Do you hold any stocks or shares in an organisation that may in any way gain or lose financially from the publication of this manuscript, either now or in the future?
- Do you hold or are you currently applying for any patents relating to the content of the manuscript?
- Have you received reimbursements, fees, funding, or salary from an organization that holds or has applied for patents relating to the content of the manuscript?
- Do you have any other financial competing interests?
- Do you have any non-financial competing interests in relation to this paper?

If you can answer no to all of the above, write 'I declare that I have no competing interests' below. If your reply is yes to any, please give details below.

I work for GigaScience where I perform checks on the accessibility of health informatics data and the analysis approach. My funding is not dependent on the outcome of this review, and I declare that I have no competing interests.

I agree to the open peer review policy of the journal. I understand that my name will be included on my report to the authors and, if the manuscript is accepted for publication, my named report including any attachments I upload will be posted on the website along with the authors' responses. I agree for my report to be made available under an Open Access Creative Commons CC-BY license (http://creativecommons.org/licenses/by/4.0/). I understand that any comments which I do not wish to be included in my named report can be included as confidential comments to the editors, which will not be published.

Choose an item.

To further support our reviewers, we have joined with Publons, where you can gain additional credit to further highlight your hard work (see: https://publons.com/journal/530/gigascience). On publication of this paper, your review will be automatically added to Publons, you can then choose whether or not to claim your Publons credit. I understand this statement.

Yes Choose an item.
