# Supplementary material for: ricu: R’s interface to intensive care data
Source: Gigascience. 2023 Jun 15;12:giad041. doi: 10.1093/gigascience/giad041 (PMC10268223; doi:10.1093/gigascience/giad041)
Supplement: giad041_Reviewer_2_Report_Original_Submission — Emre Guney, Ph.D. -- 3/1/2023 Reviewed [file giad041_reviewer_2_report_original_submission.pdf]

Reviewer Report

Title: ricu: R's Interface to Intensive Care Data

Version: Original Submission **Date:** 3/1/2023

Reviewer name: Emre Guney, Ph.D.

Reviewer Comments to Author:

Bennett et al. provide an R package (ricu) for harmonizing and analyzing data from several intensive care unit data sets, including MIMIC, eICU, AUMCdb and HiRID. The manuscript is well written and addresses and important need, i.e., facilitating access to publicly available clinical data (upon registration) for reproducible analyzes of intensive care data.My major concern is that the authors generate their own concept dictionary, mapping terms to SNOMED-CT (whenever possible) and using abbreviated version of the text. Given some of the data sets already use a standard common data model, e.g., OMOP CDM for MIMIC data sets, I find generating their own dictionary rather counter-intuitive towards their standardization effort compared to using concept dictionary from existing initiatives (which covers SNOMED-CT among other vocabularies and can be browsed at https://athena.ohdsi.org/). Therefore, I would recommend to adopt ATHENA concepts, which is likely to increase the coverage of mapped terms as well as minimize the need for generating custom concepts. I also find the use of abbreviations rather than the standardized terms confusing, I believe the package should accept the full term / standardized id (in addition to the concept abbreviation in ricu dictionary).Minor points1. It would be good to mention also in the abstract that the data is subject to request of access (or only toy/demo data available from the data sources).2. Introduction could mention related work on the R packages for analysis & visualization of OMOP data such as ROMOP and PatientExploreR. A discussion on why the authors chose to create a new package rather than converting the data from non-OMOP based data source into OMOP format and use an existing package would be useful.3. Text mentions 5 categories, however, only 4 displayed in Table 2.4. In Figure 2, for which of the amount intervals the difference between the two groups (diabetic vs non) are significant?5. Authors mention that not every concept is in SNOMED-CT, it would be good to have these terms in Supplementary and show whether they exist in ATHENA.

Level of Interest

Please indicate how interesting you found the manuscript: Choose an item.

Quality of Written English

Please indicate the quality of language in the manuscript: Choose an item.

Declaration of Competing Interests

Please complete a declaration of competing interests, considering the following questions:

- Have you in the past five years received reimbursements, fees, funding, or salary from an organisation that may in any way gain or lose financially from the publication of this manuscript, either now or in the future?
- Do you hold any stocks or shares in an organisation that may in any way gain or lose financially from the publication of this manuscript, either now or in the future?
- Do you hold or are you currently applying for any patents relating to the content of the manuscript?
- Have you received reimbursements, fees, funding, or salary from an organization that holds or has applied for patents relating to the content of the manuscript?
- Do you have any other financial competing interests?
- Do you have any non-financial competing interests in relation to this paper?

If you can answer no to all of the above, write 'I declare that I have no competing interests' below. If your reply is yes to any, please give details below.

I declare that I have no competing interests

I agree to the open peer review policy of the journal. I understand that my name will be included on my report to the authors and, if the manuscript is accepted for publication, my named report including any attachments I upload will be posted on the website along with the authors' responses. I agree for my report to be made available under an Open Access Creative Commons CC-BY license (http://creativecommons.org/licenses/by/4.0/). I understand that any comments which I do not wish to be included in my named report can be included as confidential comments to the editors, which will not be published.

Choose an item.

To further support our reviewers, we have joined with Publons, where you can gain additional credit to further highlight your hard work (see: https://publons.com/journal/530/gigascience). On publication of this paper, your review will be automatically added to Publons, you can then choose whether or not to claim your Publons credit. I understand this statement.

Yes Choose an item.
