# Supplementary material for: ricu: R’s interface to intensive care data
Source: Gigascience. 2023 Jun 15;12:giad041. doi: 10.1093/gigascience/giad041 (PMC10268223; doi:10.1093/gigascience/giad041)
Supplement: giad041_Reviewer_2_Report_Revision_1 — Emre Guney, Ph.D. -- 3/9/2023 Reviewed [file giad041_reviewer_2_report_revision_1.pdf]

Reviewer Report

Title: ricu: R's Interface to Intensive Care Data

Version: Revision 1 **Date:** 3/9/2023

Reviewer name: Emre Guney, Ph.D.

Reviewer Comments to Author:

I thank the authors for addressing my comments. I would bring up the following additional points for their consideration:- It seems the package on gitlab and CRAN are not on sync.- The devtools::install_github("eth-mds/ricu") command gives an error as there is no "master" branch (I was able to install adding a parameter referring to "main" branch)- The documentation of load_concept function does not seem to reflect the newly added functionality of loading concepts using omop id- It is unclear whether "load_dictionary" function loads all the concepts from OMOP or not only the ones that were used in ricu. I would recommend to have the functionality to load all concepts from OMOP (if not available already) which would facilitate the addition of other data sets by users (instead of creating them one-by-one as per need)- Clarify how the difference between two groups are calculated for the following statement: "(p-value = 0.04 for the independence of diabetes and insulin amount)". That is which test, alternative hypothesis & one or two-sided and what is the effect size (confidence interval)?- One would be interested seeing the difference per amount of insulin group in Figure 2 (can be annotated by a star in the figure if $P<0.05$, two stars if $P<0.01$ etc. -- with proper legend).- It would also be good to see what was the effect size & p-value for the full data set in relation to their statement "this effect is more pronounced when looking at the full MIMIC-III data".

Level of Interest

Please indicate how interesting you found the manuscript: Choose an item.

Quality of Written English

Please indicate the quality of language in the manuscript: Choose an item.

Declaration of Competing Interests

Please complete a declaration of competing interests, considering the following questions:

- Have you in the past five years received reimbursements, fees, funding, or salary from an organisation that may in any way gain or lose financially from the publication of this manuscript, either now or in the future?
- Do you hold any stocks or shares in an organisation that may in any way gain or lose financially from the publication of this manuscript, either now or in the future?

- Do you hold or are you currently applying for any patents relating to the content of the manuscript?
- Have you received reimbursements, fees, funding, or salary from an organization that holds or has applied for patents relating to the content of the manuscript?
- Do you have any other financial competing interests?
- Do you have any non-financial competing interests in relation to this paper?

If you can answer no to all of the above, write 'I declare that I have no competing interests' below. If your reply is yes to any, please give details below.

I declare that I have no competing interests

I agree to the open peer review policy of the journal. I understand that my name will be included on my report to the authors and, if the manuscript is accepted for publication, my named report including any attachments I upload will be posted on the website along with the authors' responses. I agree for my report to be made available under an Open Access Creative Commons CC-BY license (http://creativecommons.org/licenses/by/4.0/). I understand that any comments which I do not wish to be included in my named report can be included as confidential comments to the editors, which will not be published.

Choose an item.

To further support our reviewers, we have joined with Publons, where you can gain additional credit to further highlight your hard work (see: https://publons.com/journal/530/gigascience). On publication of this paper, your review will be automatically added to Publons, you can then choose whether or not to claim your Publons credit. I understand this statement.

Yes Choose an item.
